# Chemogenetic Inhibition of Lateral Hypothalamus Inputs to the Paraventricular Thalamus Reduces Goal-Tracking in a Behaviorally Flexible Subgroup of Male Rats

**DOI:** 10.1523/ENEURO.0174-26.2026

**Published:** 2026-09-04

**Authors:** Amanda G. Iglesias, Jasmine K. Bhatti, Alexandra E. Turfe, Stephen Chang, Hsin-Jui Lu, Paolo Campus, Shelly B. Flagel

**Affiliations:** ^1^Neuroscience Graduate Program, University of Michigan, Ann Arbor, Michigan 48104; ^2^Michigan Neuroscience Institute, University of Michigan, Ann Arbor, Michigan 48104; ^3^College of Literature, Science, and the Arts, University of Michigan, Ann Arbor, Michigan 48104; ^4^Department of Psychiatry, University of Michigan, Ann Arbor, Michigan 48104

**Keywords:** incentive salience, individual differences, lateral hypothalamus, motivation, paraventricular thalamus

## Abstract

The paraventricular nucleus of the thalamus (PVT) has emerged as an important node in circuits regulating motivated behavior. The neuronal pathway from the lateral hypothalamus (LH) to the PVT has been shown to regulate arousal, feeding, and reward seeking. However, the involvement of the LH–PVT pathway in individual differences in cue-motivated behavior remains unclear. During a pavlovian conditioned approach (PavCA) paradigm, when a reward is repeatedly preceded by the presentation of a cue, rats come to exhibit a conditioned response to the cue. One extreme of the population, sign-trackers (STs), approaches and interacts with the cue itself, while the other extreme, goal-trackers (GTs), approaches the location of reward delivery. Intermediate responders (IRs) approach and interact with both the cue and reward location, without a preference. We utilized a PavCA paradigm in male rats to examine the effects of LH–PVT pathway inhibition on individual differences in cue-motivated behavior. A dual-vector approach was used to express inhibitory chemogenetic receptors in the LH–PVT pathway. We found that inhibition of the LH–PVT pathway selectively attenuates the expression of goal-tracking behavior, without affecting sign-tracking. This effect is driven primarily by IR rats, as inhibition of LH–PVT neurons attenuates goal-tracking behavior in IRs, without impacting the response of STs or GTs. We speculate that the flexibility of responding in IR rats made them especially vulnerable to this manipulation. These findings identify the LH–PVT pathway as a selective contributor to reward-directed conditioned responding and a circuit substrate for behavioral flexibility.

## Significance Statement

Individuals differ in how reward-predictive cues motivate behavior, a feature linked to vulnerability and resilience to maladaptive reward seeking. The paraventricular thalamus (PVT), a midline hub with broad limbic connectivity, and its input from the lateral hypothalamus (LH) influence arousal, feeding, and reward seeking, but the role of the LH–PVT pathway in individual variability in cue-motivated behavior is unclear. We used chemogenetics to selectively suppress LH–PVT neurons during a pavlovian conditioned approach paradigm. Inhibiting this projection reduced goal-tracking without altering sign-tracking, and this effect was driven primarily by intermediate responders, a phenotype characterized by behavioral flexibility. These results implicate LH–PVT signaling in reward-directed conditioned responding and suggest that this pathway contributes to flexible cue-motivated behavior.

## Introduction

Decades of work have sought to identify neural circuits that contribute to motivated behavior and reward learning, much of it centered on the mesocorticolimbic dopamine system (Berridge and Robinson, 1998; Berridge, 2007; Bromberg-Martin et al., 2010). Yet, motivated behavior arises from a broader network of cortical and subcortical structures. Within this network, the paraventricular nucleus of the thalamus (PVT) has emerged as a node implicated in arousal (Hsu et al., 2014; Kirouac, 2015), reward seeking (Matzeu et al., 2014; Kirouac, 2015), stress responsivity (Bhatnagar and Dallman, 1998, 1999; Bhatnagar et al., 2002, 2003; Li et al., 2010b; Heydendael et al., 2011), motivational conflict (Choi and McNally, 2017; Choi et al., 2019), and cue-motivated behavior (Schiltz et al., 2007; Choi et al., 2010; Igelstrom et al., 2010; Matzeu et al., 2017; Beas et al., 2024). Foundational work by Kelley and colleagues placed the PVT within a hypothalamic–thalamic–striatal circuit relevant to motivated behavior (Kelley et al., 2005). The PVT receives inputs from cortical, hypothalamic, and brainstem regions and projects to limbic targets including the nucleus accumbens (NAc), bed nucleus of the stria terminalis, and central amygdala (Li and Kirouac, 2008; Hsu and Price, 2009). This connectivity positions the PVT to integrate arousal- and state-related signals with environmental stimuli to influence motivated behavior (Kirouac, 2015; Barson et al., 2020; Iglesias and Flagel, 2021; Penzo and Gao, 2021).

Among subcortical inputs to the PVT, the lateral hypothalamus (LH) is of particular interest (Kirouac et al., 2005; Kirouac, 2015). The LH has long been linked to appetitive and consummatory processes (Hoebel and Teitelbaum, 1962; Margules and Olds, 1962), including findings that LH stimulation elicits intense feeding (Anand and Brobeck, 1951; Delgado and Anand, 1953). However, LH is now recognized as a heterogeneous structure supporting functions beyond feeding and energy balance (Bonnavion et al., 2016; Stuber and Wise, 2016; Petrovich, 2018; Chen et al., 2025). LH inputs to the PVT include orexinergic and other neurochemically diverse populations implicated in feeding, arousal, and responses to salient sensory stimuli (Kirouac et al., 2005; Ren et al., 2018; Otis et al., 2019). Yet, the contribution of the LH–PVT pathway to pavlovian cue-motivated behavior remains unclear.

A pavlovian conditioned approach (PavCA) paradigm can capture individual variability in conditioned responses (CR) to reward-paired cues (Flagel et al., 2007; Robinson and Flagel, 2009; Meyer et al., 2012; Hughson et al., 2019) and helps elucidate mechanisms underlying cue-motivated behavior (Flagel et al., 2011a,b; Campus et al., 2019; Haight et al., 2020). In this procedure, a lever-cue (conditioned stimulus, CS) precedes food reward delivery (unconditioned stimulus, US), and animals develop distinct CR directed toward the cue itself (sign-tracking) or the site of reward delivery (goal-tracking). For sign-trackers (STs), the lever-cue acquires both predictive and incentive value, whereas for goal-trackers (GTs), it primarily acquires predictive value (Robinson and Flagel, 2009). The attribution of incentive motivational value or incentive salience transforms the cue into an attractive and desirable stimulus capable of controlling behavior in STs (Robinson and Berridge, 1993; Berridge et al., 2009; Flagel et al., 2009). Intermediate responders (IRs) show greater behavioral flexibility, vacillating between sign- and goal-tracking.

Prior work has identified the PVT as an important node regulating sign- and goal-tracking behaviors (Flagel et al., 2011a; Haight and Flagel, 2014; Haight et al., 2015; Haight et al., 2017; Kuhn et al., 2018; Campus et al., 2019). A top–down corticothalamic pathway has been proposed to encode the predictive value of reward cues and regulate conditioned responding across phenotypes, whereas bottom–up inputs to the PVT are thought to relay the incentive value of reward-associated cues (Haight and Flagel, 2014; Campus et al., 2019). Within this framework, the LH is of particular interest, as it sends dense orexinergic projections to the PVT (Peyron et al., 1998; Kirouac et al., 2005). Consistent with this idea, LH lesions attenuate the development of sign-tracking behavior, and pharmacologically blocking orexin signaling in the PVT reduces sign-tracking and the incentive motivational value of the lever-cue (Haight et al., 2020).

Here, we examined the role of the LH–PVT pathway in the expression of cue-motivated behavior in male rats using chemogenetics. We assessed the effects of LH–PVT inhibition across the population and by the PavCA phenotype. Based on prior work, we hypothesized that inhibiting LH–PVT projections would reduce sign-tracking by diminishing the incentive value of the cue. Instead, LH–PVT inhibition selectively attenuated goal-tracking, an effect driven by IRs, with no detectable effect within STs or GTs.

## Materials and Methods

Behavioral testing took place during the light-phase between 10 A.M. and 4 P.M. All procedures followed *The Guide for the Care and Use of Laboratory Animals: Eighth Edition* (2011, National Academy of Sciences) and were approved by the University of Michigan Institutional Animal Care and Use Committee. Rats were run in two separate experiments. Experiment 1 assessed the effect of chemogenetic inhibition of the LH–PVT pathway on PavCA behavior in rats expressing inhibitory DREADDs (designer receptors exclusively activated by designer drugs). Experiment 2 served as a no-DREADD control experiment used to determine whether clozapine-*N*-oxide (CNO; DREADD ligand) in the absence of DREADD, affected PavCA behavior. In Experiment 1, rats underwent surgery to express DREADDs in the LH–PVT pathway and were run across multiple rounds, with either 30 or 40 rats tested per round. In Experiment 2, rats did not undergo surgery or express DREADDs and were run in one round of 60 rats.

**Figure 1.**
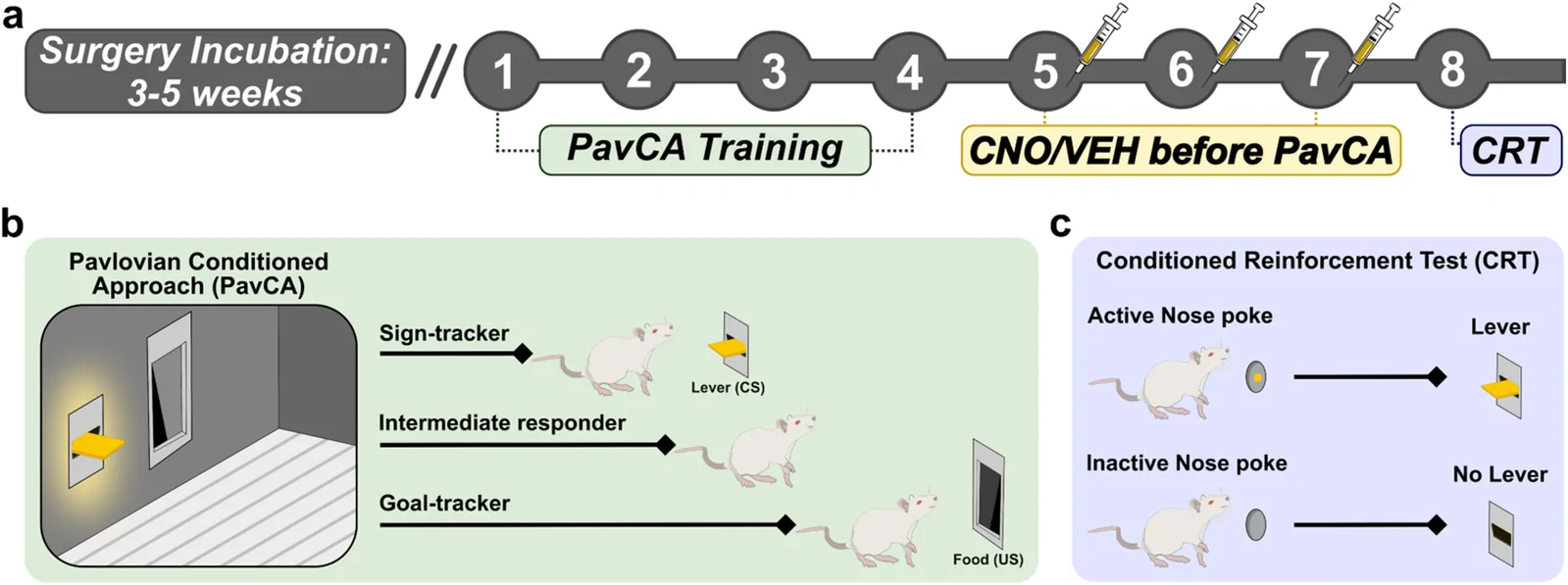
Experimental timeline. ***a***, Schematic of the experimental timeline: Stereotaxic surgery was performed to infuse inhibitory DREADD virus in the LH to the PVT pathway. The virus incubated in the brain for 3–5 weeks and was followed by behavioral assays. PavCA training occurred for 7 consecutive days. On Days 5–7 of behavior, either CNO (5 mg/kg) or vehicle (VEH; 6% DMSO) was injected intraperitoneally. On Day 8 of behavior rats had a CRT with no injections immediately prior. ***b***, PavCA schematic illustrating the behavior box and responses of ST, IR, and GT. ***c***, CRT schematic wherein nose pokes in the port designated active result in lever presentation and those in the inactive port are without consequence.

The behavioral timeline was the same for both experiments (Fig. 1). Rats underwent seven consecutive sessions of PavCA. We aimed to test the effects of LH–PVT pathway inhibition during the expression of PavCA behavior. After Sessions 3–4 of PavCA (acquisition), rats were given vehicle (VEH) injections to acclimate them to receiving injections. Twenty-five minutes prior to Sessions 5–7 of PavCA (expression), rats were given either VEH or CNO. On Session 8, all rats underwent a conditioned reinforcement test (CRT), with no injections given prior to testing.

### Subjects

Rats were ∼9 weeks of age (∼300–350 g body weight) at the time of arrival and were housed under a 12 h light/dark cycle (lights on at 6 or 7 A.M. depending on daylight saving time) with climate-controlled conditions (22 ± 2°C). They had *ad libitum* access to food and water throughout the study. Rats were pair-housed for the duration of the experiment and were acclimated to the housing room for a minimum of 1 week before experimenter handling began.

Male Sprague Dawley rats (*N* = 230 in total) were ordered from different barriers at either Charles River Laboratories or Taconic Biosciences. In Experiment 1 (five rounds, *n* = 170), rats were from Charles River barrier R04 (*n* = 41), Charles River barrier R08 (*n* = 64), Taconic BU16 (*n* = 20), or Taconic IBU (*n* = 45). Seven rats were removed from the study before behavioral testing due to surgical or postsurgical complications. Rats were characterized as STs, IRs, or GTs, based on PavCA behavior on Session 4, prior to any CNO administration. Rats were then assigned to VEH or CNO treatment groups in a counterbalanced manner within each phenotype. To avoid overrepresentation of a single phenotype within a given round, rats (*n* = 66) from overrepresented phenotypes were randomly excluded prior to DREADD activation and before any test-phase behavior was analyzed. Additionally, rats (*n* = 43) were excluded for off-target viral expression or minimal expression in the LH, aPVT, pPVT, or bilaterally across the targeted pathway (see below, Immunohistochemistry). These behavioral and anatomical exclusions were applied in sequence and contributed to the final sample sizes. A total of *n* = 54 rats were included in Experiment 1, as follows: STs (*n* = 13; 7 VEH/6 CNO), IRs (*n* = 18; 7 VEH/11 CNO), or GTs (*n* = 23; 15 VEH/8 CNO). In Experiment 2, the no-DREADD cohort (*n* = 60), rats were either from Charles River barrier R04 (*n* = 30) or Charles River barrier R08 (*n* = 30). We ordered from Charles River barriers for this cohort because, historically, these barriers yielded a more even distribution across phenotypes. A total of *n* = 60 rats were used for Experiment 2, as follows: STs (*n* = 21; 11 VEH/10 CNO), IRs (*n* = 23; 11 VEH/12 CNO), or GTs (*n* = 16; 8 VEH/8 CNO).

### Viral vectors

Viruses were obtained from Addgene. The Cre-dependent *G_i_* DREADD virus (AAV8-hSyn-DIO-hM4Di-mCherry, Addgene 44362-AAV8) had a titer of ≥1 × 10^13^ vg/ml. The Cre virus (AAVrg-pmSyn1-EBFP-Cre, Addgene 51507-AAVrg) had a titer of ≥7 × 10^12^ vg/ml.

### DREADD ligand

We obtained CNO from the National Institute of Mental Health. DREADD activation occurred via systemic administration of CNO, which results in inhibition of the LH–PVT pathway through disruption of G-protein–dependent signaling, likely from induced hyperpolarization (Armbruster et al., 2007; Vardy et al., 2015) or inhibition of presynaptic release of neurotransmitters (Stachniak et al., 2014; Vardy et al., 2015). CNO (5 mg/kg) was dissolved in 6% dimethyl sulfoxide (DMSO) and sterile H_2_O and injected intraperitoneally (Armbruster et al., 2007; Ferguson et al., 2011; Rogan and Roth, 2011; Stachniak et al., 2014). Control animals were administered vehicle intraperitoneally (VEH; 6% DMSO and sterile H_2_O).

### Surgery

Surgeries were performed using a dual-vector approach to selectively express hM4Di-DREADDs (*G_i_*, inhibitory) in the LH–PVT pathway. Rats were anesthetized with 5% isoflurane (Vet One) delivered via an induction chamber and were then placed into the stereotaxic frame (David Kopf Instruments or Stoelting) and given an injection of carprofen (5 mg/kg, s.c.) for analgesia. Rats remained at 2% isoflurane for anesthetic maintenance. Surgeries were performed under aseptic conditions. All coordinates were relative to the bregma (Paxinos and Watson, 2007). A Neuros Syringe (5 µl Model 33 gauge, point style 3, Hamilton Company) infused 0.75 µl of an inhibitory Cre-dependent Gi DREADD virus bilaterally into the LH [−2.7 mm AP (anterior/posterior); ±1.6 mm ML (medial/lateral); −9 mm DV (dorsal/ventral)]. The DREADD virus was infused over 8 min, and the syringe was left in place for an additional 10 min. A Neuros Syringe (0.5 µl Model 33 gauge, point style 3, Hamilton Company) infused 0.10 µl of a retrograde Cre virus at a 10° angle into the anterior PVT (aPVT, −2.0 mm AP; −1 mm ML; −5.4 mm DV) and posterior PVT (pPVT, −3.0 mm AP; −1 mm ML; −5.5 mm DV) to target the entire rostrocaudal axis of the region. The Cre virus was infused over 2 min, and the syringe was left in place for an additional 5 min. Following 3–5 weeks of virus incubation, all rats underwent PavCA training.

### PavCA

PavCA training occurred inside Med Associates chambers (24.1 × 21 × 30.5 cm) located in sound-attenuating boxes equipped with a fan to reduce background noise. The chambers contained a food cup that was connected to a pellet dispenser and placed in the center of one wall 3 cm above the chamber floor. An illuminated retractable lever-cue was located either to the left or right of the food cup, 6 cm above the chamber floor. A white house light was placed at the top of the chamber on the wall opposite the food cup and lever-cue and remained on for the duration of the session. Food cup entries were recorded upon a photo-beam break inside the food cup, and a lever-cue contact was recorded following a minimum deflection force of 10 g.

All rats were handled by experimenters for 2 d before assessing PavCA behavior. Rats were not food restricted and had *ad libitum* access to home cage chow and water throughout behavioral testing. For the 2 d prior to pretraining, rats received ∼25 banana-flavored grain pellets (each pellet 45 mg; Bio-Serv) in their home cage to familiarize them with the reward. Prior to each session, the rats were transferred to the injection room 1 h prior to behavior. Rats were initially placed into the Med Associates chambers for a single pretraining session. At the start of the pretraining session, the food cup was baited with two food pellets to direct the rats' attention to the location of reward delivery. During pretraining, the lever-cue remained retracted, and rats received a food pellet in the food cup on a variable 30 s (range 0–60 s) schedule. There were 25 trials, and the pretraining session lasted ∼12.5 min, during which food cup entries were recorded, and food pellet consumption was confirmed. Following pretraining, rats had a single session of PavCA each day for 7 consecutive days (Fig. 1*a*,*b*). The acquisition phase of PavCA behavior occurred across the first four sessions. Following behavior on Sessions 3–4, rats received intraperitoneal VEH injections to habituate them. Rats were assigned to phenotype and treatment groups in a counterbalanced manner based on their behavior on Session 4. On Sessions 5–7, rats received intraperitoneal VEH or CNO injections 25 min before the start of the session each day. Each PavCA session began with a 5 min waiting period, followed by illumination of the house light to signify the start of the session. During PavCA, the illuminated lever-cue (CS) entered the chamber for 8 s, and upon retraction, a food pellet (US) was immediately delivered into the adjacent food cup. PavCA sessions consisted of 25 lever-cue (CS)/food-US trials on a variable 90 s schedule (range 30–150 s; Meyer et al., 2012; Campus et al., 2019). Each session lasted ∼40 min. It was confirmed that all food pellets had been consumed following each session.

Med Associates software recorded the following behaviors during PavCA sessions: (1) number of food cup entries made during the 8 s lever presentation, (2) latency to contact the food cup during lever-cue presentation, (3) number of lever-cue contacts, (4) latency to contact the lever-cue, and (5) the number of food cup entries made during the intertrial interval (ITI; i.e., entries made between lever-cue presentations). Contact, latency, and probability data were used to calculate the daily PavCA score, and the score from Session 4 was used to categorize rats into their behavioral phenotypes (Meyer et al., 2012). The PavCA score is a composite measure calculated using the following formula: [(Probability Difference Score + Response Bias Score + (−Latency Difference Score))/3]. PavCA score values range from −1 to 1, with −1 representing individuals with a CR focused solely on the food cup during lever-cue presentation (i.e., “pure” GTs) and +1 representing individuals with a CR focused solely on the lever-cue upon its presentation (i.e., “pure” STs). IRs vacillate between the two responses. The PavCA Index was generated from the behavioral measures during Session 4 to classify animals as STs (PavCA Index ≥ 0.5), IRs (−0.5 < PavCA Index < 0.5), or GTs (PavCA Index ≤ −0.5). Prior studies have reported that PavCA phenotypes are generally stable at this stage of training (Campus et al., 2019). Thus, we used data from Session 4 for phenotype classification because it was the final acquisition session prior to CNO administration and therefore provided the last premanipulation assessment of conditioned responding. This allowed VEH and CNO treatment groups to be assigned across phenotypes in a counterbalanced manner.

### CRT

Rats were exposed to a CRT the day following the final PavCA session (Fig. 1*a*,*c*). This test required rats to perform an instrumental response for lever presentation, in the absence of reward (Robinson and Flagel, 2009). That is, the lever that was previously the reward cue now served as the reinforcer. Conditioning chambers were reconfigured, and the lever was placed in the center wall, flanked by nose ports. Nose pokes into the “active” port resulted in presentation of the illuminated lever for 2 s, while nose pokes into the “inactive” port had no consequence. The active port was placed opposite the side of the lever location during PavCA sessions to minimize side bias. The CRT lasted 40 min. The Med Associates software recorded the number of nose pokes into the “active” and “inactive” ports and the number of contacts with the lever during its brief presentation.

### Perfusion and tissue processing

Rats that previously received surgery were perfused within 5 d following the experiment. Rats were anesthetized with a cocktail of ketamine (90 mg/kg, i.p.) and xylazine (10 mg/kg, i.p.). Ten minutes after the anesthetic injection, rats were transcardially perfused with 0.9% saline and 4% formaldehyde, pH 7.4. Following brain extraction, the tissue was postfixed in 4% formaldehyde for 24 h at 4°C and then placed in 30% sucrose at 4°C (sucrose in 0.1 M PBS), pH 7.4, for 3 d. The brains were frozen using dry ice and coated in Tissue-Plus optimal cutting temperature (OTC) compound (Fisher HealthCare). Serial coronal brain slices were taken at 40 µm using a cryostat at −20°C (Leica Biosystems).

### Immunohistochemistry

DREADD expression in LH–PVT projection neurons was evaluated following immunohistochemical staining of mCherry, the fluorescent tag on the *G_i_* DREADD virus. Free-floating coronal sections from the LH and PVT were initially washed six times in 0.1 M PBS, pH 7.4, to remove OTC debris. Each step after this initial wash was followed by three washes in 0.1 M PBS, pH 7.4. Sections were incubated in 1% H_2_O_2_ for 10 min to block endogenous peroxidase activity and then incubated in 0.1 M PBS containing 0.4% Triton X-100 (TX) and 2.5% normal donkey serum (NDS; Jackson ImmunoResearch Laboratories) to block nonspecific binding of the secondary antibody. Subsequently, sections were incubated overnight at room temperature in primary antibody (rabbit anti-mCherry, Abcam, diluted 1:30,000) in 0.1 M PBS + 0.4% TX + 1% NDS, and the next-day sections were incubated for 1 h in a biotinylated donkey anti-rabbit secondary antibody (Jackson ImmunoResearch Laboratories, diluted 1:500) in 0.1 M PBS + 0.4% TX + 1% NDS. Sections were then incubated for 1 h in Vectastain Elite ABC solution (1:1,000 A and 1:1,000 B, diluted in 0.1 M PBS, mixed 30 min before use; Vector Laboratories). This stain was visualized by incubating the sections in 0.1 M NaPB containing 0.02% 3,3′-diaminobenzidine tetrahydrochloride (DAB; Sigma-Aldrich) and 0.012% H_2_O_2_ for 10 min causing a brown precipitate to form at the location of mCherry detection. Sections were stored at 4°C until mounted, air-dried, and coverslipped with Permount (Thermo Fisher Scientific). Bright-field images containing the LH, aPVT, and pPVT were captured using a Leica DM1000 light microscope (Leica Microsystems) and were analyzed by an experimenter blind to the experimental groups.

**Figure 2.**
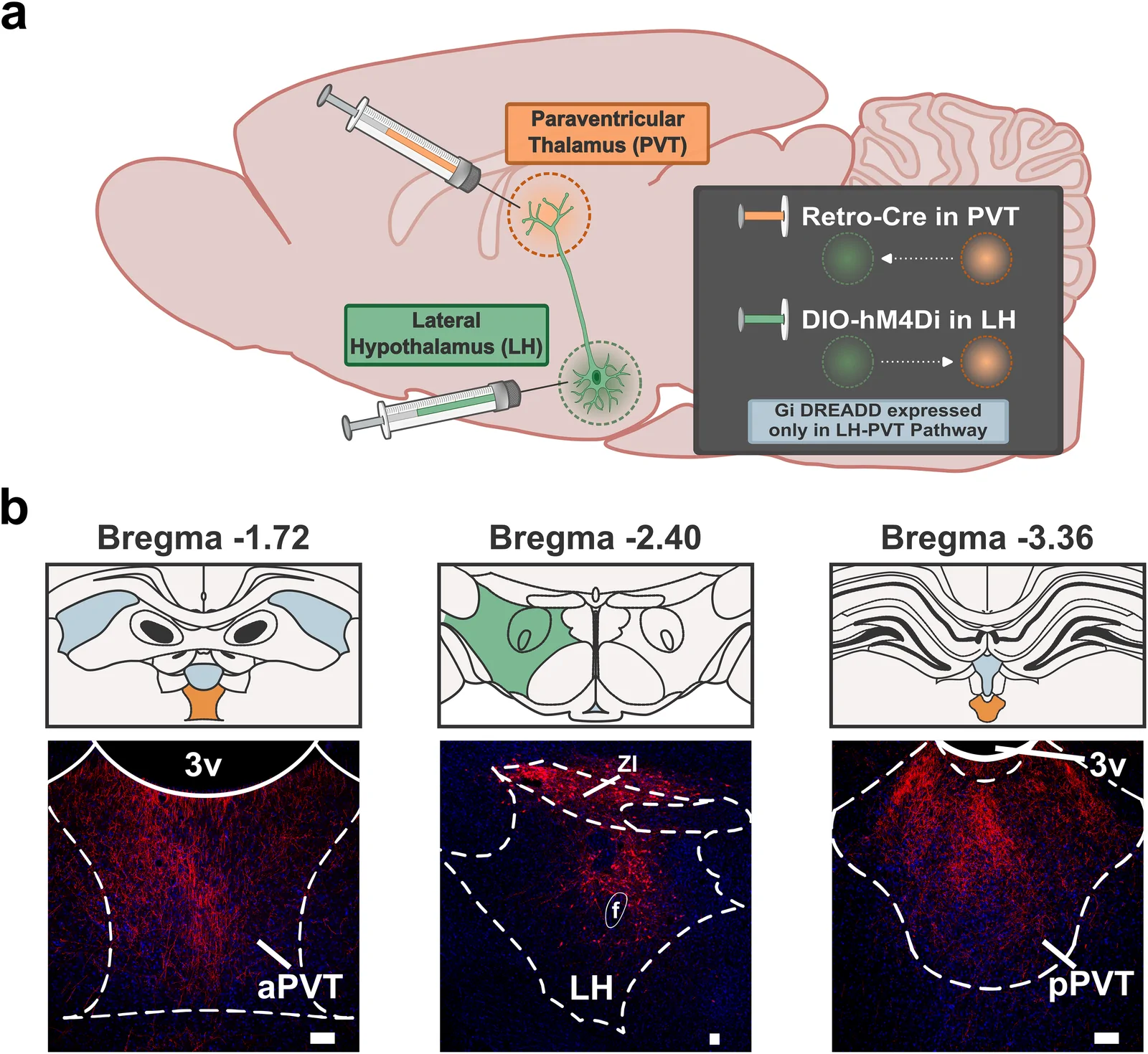
Surgery schematic and virus expression. ***a***, Schematic of the dual-vector strategy used to selectively express inhibitory DREADD in the LH–PVT pathway. A retrograde Cre virus was infused into the aPVT and pPVT, and a Cre-dependent inhibitory DREADD virus was infused bilaterally into the LH. ***b***, Representative images showing DREADD virus expression in the aPVT (10× magnification), LH (4× magnification), and pPVT (10× magnification). Scale bars in the bottom right corner represent 100 µm. 3v, third ventricle; ZI, zona incerta; f, fornix.

The DAB-processed tissue was used for anatomical assessment, expression scoring, and inclusion/exclusion decisions. An experimenter blind to group assignments gave a score of 0–3 to each image according to both the density and location of DREADD expression in the areas of interest (Fig. 2). A score of 0 was assigned to subjects that had no DREADD expression in the LH, aPVT, and pPVT or off-target DREADD expression (e.g., expression outside regional boundaries); a score of 1 was assigned to subjects that had visible DREADD expression in both the LH and PVT; a score of 2 was assigned to subjects with visible DREADD expression in one region and strong DREADD expression in the other; a score of 3 was assigned to subjects that had strong DREADD expression in both the LH and PVT. Rats that had a score of 0 (*n* = 43), meaning they had no visible expression in either the LH or PVT, were excluded from the statistical analysis. Because the LH and zona incerta (ZI) are anatomically adjacent, some mCherry-positive cell body expression was observed in the ZI in included subjects. This expression was unavoidable given the proximity of the targeted LH injection site to the ZI. Rats were excluded only when viral expression was absent from the LH/PVT pathway or when expression was judged to be off-target relative to the intended LH–PVT targeting strategy.

A subset of brains was processed for immunofluorescence to obtain publication-quality representative images of mCherry expression in the LH and PVT. Free-floating coronal sections from the LH and PVT were initially washed six times in 0.1 M PBS, pH 7.4, to remove OTC debris. Each step after this initial wash was followed by three washes in 0.1 M PBS, pH 7.4. Sections were incubated overnight at room temperature in primary antibody (rabbit anti-mCherry, Abcam, diluted 1:500) in 0.1 M PBS + 0.4% TX + 2.5% NDS. The next-day sections were incubated for 2 h in a biotinylated donkey anti-rabbit secondary antibody (Jackson ImmunoResearch Laboratories, diluted 1:500) in 0.1 M PBS + 0.4% TX + 2.5% NDS. Sections were then incubated for 1 h in Alexa Fluor 594-conjugated streptavidin (Thermo Fisher Scientific, diluted 1:1,000) in 0.1 M PBS + 0.4% TX and then mounted onto slides and coverslipped with ProLong Gold Antifade Mountant (Thermo Fisher Scientific). Fluorescent images of the LH, aPVT, and pPVT were captured using a FV3000 confocal microscope and the FV31S-SW Viewer software (OLYMPUS Microscopes; Fig. 2*b*).

### Statistical analyses

Statistical analyses were conducted with the Statistical Package for the Social Sciences program version 27.0 (SPSS, IBM). To assess PavCA behavioral outcome measures across sessions, a linear mixed-effects model (LMM) with restricted maximum likelihood estimation was used to account for repeated measures. This analysis applied multiple covariance structures to the dataset, and the structure with the lowest Akaike information criterion was selected as best fit (Verbeke, 1997; Duricki et al., 2016). When two variables were compared at a single timepoint or across two timepoints, ANOVAs were performed, as described below. For all analyses, statistical significance was set at *p* < 0.05, and Bonferroni’s post hoc comparisons were made when significant main effects or interactions were detected. The effect size (Cohen's *d*; Cohen, 1988) was calculated for pairwise comparisons. Effect sizes were considered with respect to the following indices: 0.2, small; between 0.5 and 0.8, medium; and between 1.2 and 2.0, large (Cohen, 1988; Sawilowsky, 2009).

A LMM was conducted to compare treatment (VEH vs CNO) and phenotype (ST vs IR vs GT) across PavCA sessions, with Sessions 1–4 or 5–7 used as the repeated variable. To ensure that subjects were counterbalanced between experimental groups after PavCA training, the PavCA Index from Session 4 was analyzed using a two-way ANOVA with treatment (VEH vs CNO) and phenotype (ST vs IR vs GT) as independent variables. A two-way repeated-measures ANOVA was conducted when Session 4 (“pretest”) was directly compared with the average of Sessions 5–7 (“test”), with session (“pretest” vs “test”) as the within-subject independent variable and treatment (VEH vs CNO) and phenotype (ST vs IR vs GT) as the between-subject independent variables. For both the LMM and ANOVAs, PavCA Index, ITI food cup entries, sign-tracking behaviors (number of lever-cue contacts, probability to approach the lever-cue, latency to approach the lever-cue), and goal-tracking behaviors (food cup entries during lever-cue presentation, probability to approach the food cup during lever-cue presentation, latency to approach the food cup during lever-cue presentation) were used as dependent variables.

A mixed ANOVA was used to assess the effects of treatment (VEH vs CNO), port (active vs inactive), and phenotype (ST vs IR vs GT) on nose pokes during the CRT, and an ANOVA was used to assess the effects of treatment (VEH vs CNO) and phenotype (ST vs IR vs GT) on lever contacts.

## Results

### Acquisition of PavCA behavior (pretest)

**Figure 3.**
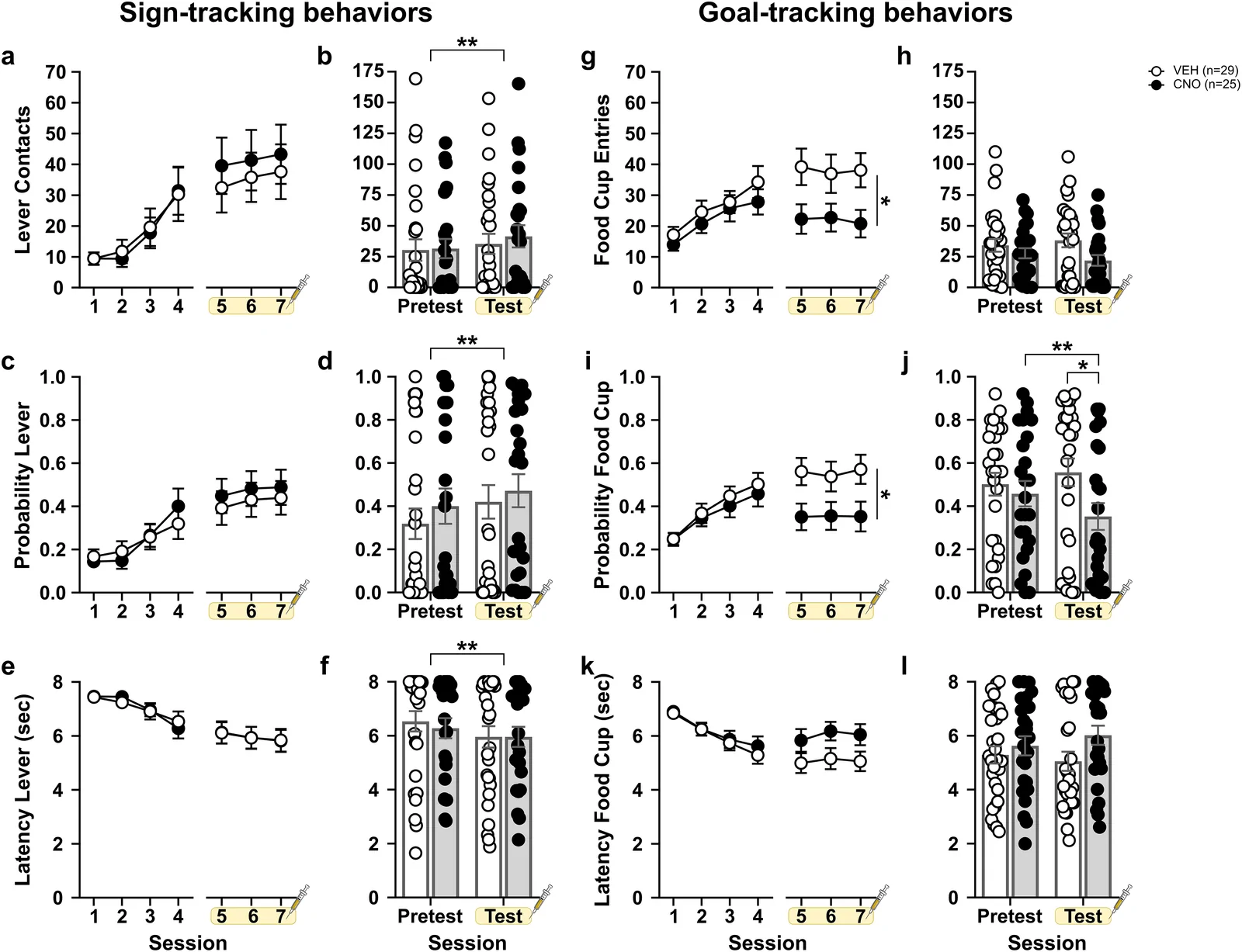
Chemogenetic inhibition of the LH–PVT pathway does not affect sign-tracking behavior but attenuates goal-tracking behavior. ***a–f***, Sign-tracking and (***g–l***) goal-tracking behaviors expressed as mean ± SEM for (top row) number of contacts/entries, (middle row) probability to approach, and (bottom row) latency to approach the lever-cue or food cup shown across PavCA Sessions 1–7 (Columns 1 and 3) and as pretest (Session 4) versus test (average of Sessions 5–7) sessions (Columns 2 and 4). On Sessions 5–7, rats received either VEH or CNO prior to testing. ***a–f***, There were no treatment effects on sign-tracking behavior. When treatment groups were collapsed, (***b***) rats made more lever-cue contacts, (***d***) showed a greater probability of approaching the lever-cue, (***f***) and had decreased latency to approach the lever-cue during test sessions relative to pretest. ***g, h***, In contrast, CNO-treated rats showed reduced goal-tracking behavior, reflected by (***g***) fewer food cup entries and (***i***) a lower probability of approaching the food cup relative to VEH-treated rats. Graphs in ***b***, ***d***, ***f***, ***h***, ***j***, and ***l*** include individual data points for all rats. Brackets indicate significant differences between or within pretest and test sessions; *p < 0.05; **p < 0.01. Associated PavCA score and pretreatment analyses are shown in Extended Data Figures 3-1 and 3-2.

The acquisition phase for PavCA occurred across Sessions 1–4. The PavCA Index on Session 4 was used to assign phenotypes and counterbalance treatment groups. There were no significant differences in the PavCA score between treatment groups during Sessions 1–4 nor in the PavCA Index on Session 4 (Extended Data Figs. 3-1*a,b*, 3-2). The PavCA score changed across Sessions 1–4 (effect of session, *F*_(3,73.871)_ = 3.164; *p* = 0.029; Extended Data Fig. 3-1*a*), but the lack of treatment effect indicated that the groups were equally balanced prior to DREADD activation. When the sign- and goal-tracking metrics that comprise the composite PavCA Index were assessed separately (Fig. 3), treatment groups learned the cue–reward relationship at similar rates across Sessions 1–4, as there was a significant effect of session for all measures, but no effect of treatment (Table 1; Fig. 3*a*,*c*,*e*,*g*,*i*,*k*). Post hoc comparisons revealed that engagement with the lever-cue and food cup significantly increased from Session 1 to Session 4 for all measures (*p* < 0.001). Thus, rats assigned to VEH and CNO groups acquired PavCA behavior similarly before any chemogenetic manipulation occurred.

10.1523/ENEURO.0174-26.2026.f3-1 Figure 3-1 **Acquisition of Pavlovian Conditioned Approach (PavCA) prior to treatment.** Data are expressed as mean ± SEM for PavCA Score (a,c) over sessions 1–4 and (b,d) on session 4 (pretest). There are no significant differences between treatment or phenotype/treatment groups in (a,c) PavCA Score across sessions 1–4 or (b,d) PavCA Index on the pretest session. (c,d) There are the expected differences between phenotypes for PavCA Score such that STs have a higher score than IRs and GTs by session 4. Download Figure 3-1, TIF file.

10.1523/ENEURO.0174-26.2026.f3-2 Figure 3-2 **Statistical analyses for PavCA Score across sessions 1**–**4.** Data from linear mixed effects model analyses for PavCA Score on sessions 1–4. The left panel compares rats by treatment, collapsed across phenotype. The right panel compares rats with treatment and phenotype as variables. Significant effects and interactions are bolded. These data correspond to data shown in Figure 3-1. Download Figure 3-2, DOCX file.

**Table 1.** Statistical analyses for effect of treatment on sign- and goal-tracking behaviors

	Sessions 1–4: sign-tracking behaviors								
	Lever contacts			Probability lever			Latency lever		
	DF	*F*	*p*	DF	*F*	*p*	DF	*F*	*p*
Session	3,62.664	7.647	**<0.001**	3,68.418	7.703	**<0.001**	3,62.467	8.544	**<0.001**
Treatment	1,52.222	0.014	0.907	1,52.491	0.008	0.929	1,52.285	0.000	0.984
Session × treatment	3,62.664	0.358	0.784	3,68.418	0.972	0.411	3,62.467	1.093	0.359
	Sessions 5–7: sign-tracking behaviors								
	Lever contacts			Probability lever			Latency lever		
	DF	*F*	*p*	DF	*F*	*p*	DF	*F*	*p*
Session	2,95.247	0.868	0.423	2,59.794	1.634	0.204	2,93.324	1.145	0.323
Treatment	1,52.028	0.252	0.618	1,52.570	0.228	0.635	1,52.066	0.000	1.000
Session × treatment	2,95.247	0.038	0.963	59.794	0.009	0.991	2,93.324	0.010	0.990
	Pretest versus test: sign-tracking behaviors								
	Lever contacts			Probability lever			Latency lever		
	DF	*F*	*p*	DF	*F*	*p*	DF	*F*	*p*
Session	1,52	7.506	**0.008**	1,52	8.604	**0.005**	1,52	10.417	0**.002**
Treatment	1,52	0.098	0.756	1,52	0.401	0.530	1,52	0.062	0.805
Session × treatment	1,52	0.847	0.362	1,52	0.260	0.612	1,52	0.839	0.364
	Sessions 1–4: goal-tracking behaviors								
	Food cup entries			Probability food cup			Latency food cup		
	DF	*F*	*p*	DF	*F*	*p*	DF	*F*	*p*
Session	3,55.853	7.157	**<0.001**	3,53.899	11.957	**<0.001**	3,68.593	15.438	**<0.001**
Treatment	1,57.616	0.996	0.322	1,58.158	0.347	0.558	1,56.378	0.217	0.643
Session × treatment	3,55.853	0.246	0.864	3,53.899	0.132	0.941	3,68.593	0.171	0.916
	Sessions 5–7: goal-tracking behaviors								
	Food cup entries			Probability food cup			Latency food cup		
	DF	*F*	*p*	DF	*F*	*p*	DF	*F*	*p*
Session	2,69.444	0.159	0.853	2,103.058	0.225	0.799	2,51.585	0.928	0.402
Treatment	1,52.137	5.147	**0.027**	1,52.025	5.119	**0.028**	1,52.012	3.655	0.061
Session × treatment	2,69.444	0.253	0.777	2,103.058	0.362	0.697	2,51.585	0.216	0.807
	Pretest versus test: goal-tracking behaviors								
	Food cup entries			Probability food cup			Latency food cup		
	DF	*F*	*p*	DF	*F*	*p*	DF	*F*	*p*
Session	1,52	0.126	0.724	1,52	1.044	0.312	1,52	0.249	0.620
Treatment	1,52	3.072	0.086	1,52	2.363	0.130	1,52	1.970	0.166
Session × treatment	1,52	3.487	0.067	1,52	10.532	**0.002**	1,52	3.857	0.055

Data from LMM analyses for Sessions 1–4 and Sessions 5–7 or a two-way repeated-measures ANOVA pretest versus test sessions with comparisons by treatment, collapsed across phenotype. Contacts, probability, and latency are represented for (top) sign-tracking behaviors and (bottom) goal-tracking behaviors. Significant effects and interactions are bolded. These data correspond to that shown in Figure 3.

**Figure 4.**
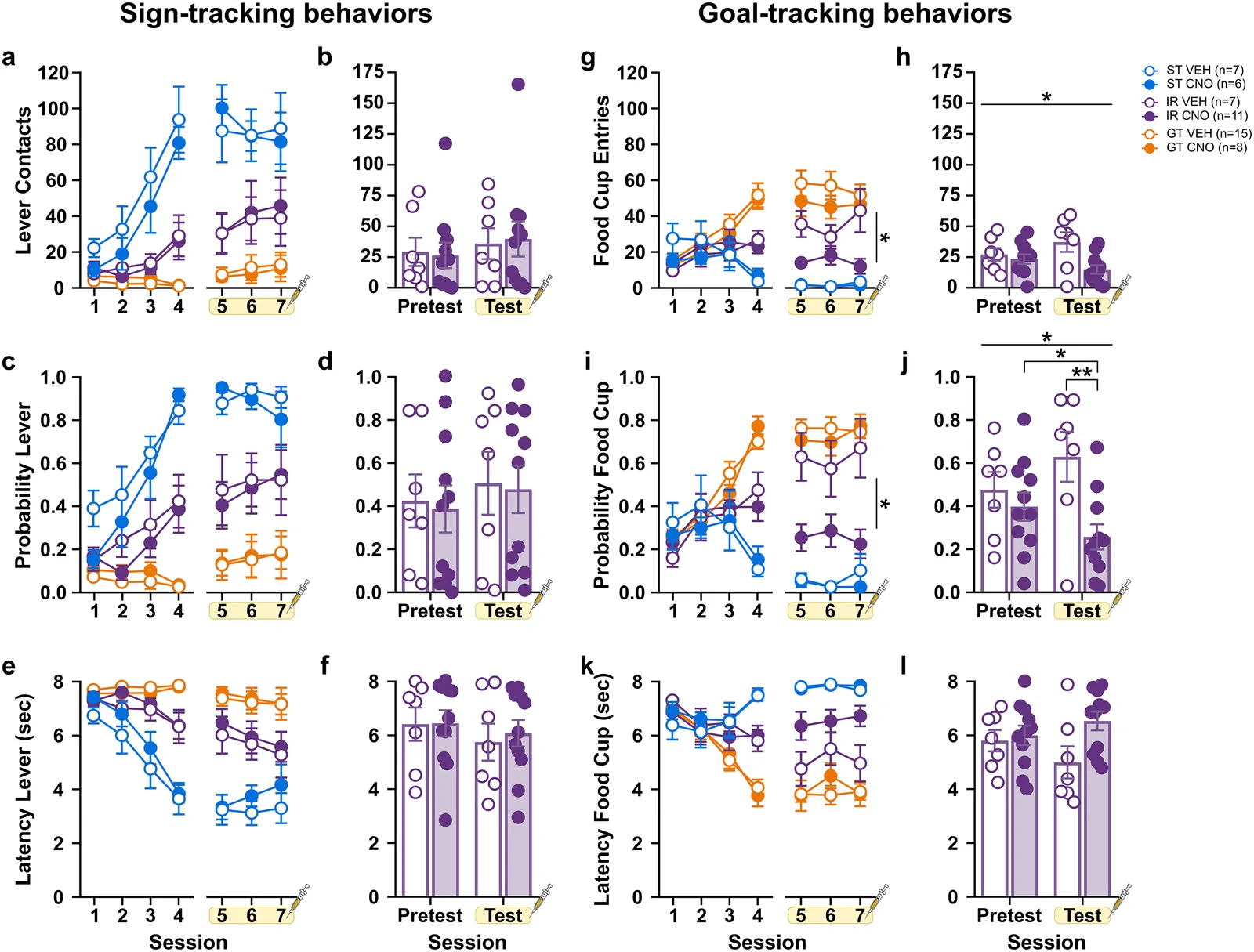
Chemogenetic inhibition of the LH–PVT pathway attenuates goal-tracking behavior in IRs. ***a–f***, Sign-tracking behaviors and (***g–l***) goal-tracking behaviors are expressed as mean ± SEM for (top row) number of contacts/entries, (middle row) probability to approach, and (bottom row) latency to approach the lever-cue or food cup across PavCA Sessions 1–7 (Columns 1 and 3) and as pretest (Session 4) versus test (average of Sessions 5–7) sessions (Columns 2 and 4). On Sessions 5–7, rats received either VEH or CNO prior to testing. ***a–f***, There were no treatment effects on sign-tracking behavior within any of the phenotypes. ***g–h***, In contrast, CNO-treated IRs showed attenuated goal-tracking behavior, reflected by (***g***) fewer food cup entries and (***j***) a lower probability of approaching the food cup relative to VEH-treated IRs. No treatment effects were observed in STs or GTs on goal-tracking indices. Graphs in ***b***, ***d***, ***f***, ***h***, ***j***, and ***l*** show pretest versus test comparisons and include individual data points for IR rats. Straight lines indicate a significant main effect of treatment. Brackets indicate significant differences between or within pretest and test sessions; *p < 0.05; **p < 0.01. Associated analyses of ITI food cup entries, conditioned reinforcement, and no-DREADD CNO controls are shown in Extended Data Figures 4-1–4-10.

When phenotype was incorporated into the analysis for PavCA score, there were no significant effects of treatment, but there were significant differences between phenotypes, as expected (effect of session, *F*_(3,65.959)_ = 3.614; *p* = 0.018; effect of phenotype, *F*_(2,50.528)_ = 42.631; *p* < 0.001; session × phenotype interaction, *F*_(6,65.959)_ = 20.242; *p* < 0.001; Extended Data Fig. 3-1*c*). In agreement, the treatment groups within each respective phenotype had similar rates of sign-tracking (Table 2; Fig. 4*a*,*c*,*e*) and goal-tracking (Table 3; Fig. 4*g*,*i*,*k*) behaviors, evidenced by significant effects of session and phenotype as well as a significant session × phenotype interaction for all measures, but no effect of treatment (Tables 2, 3). Post hoc comparisons revealed that, by Session 4, STs, IRs, and GTs were all significantly different from one another across all sign-tracking (*p* < 0.001) and goal-tracking measures (*p* < 0.001) and on the composite PavCA Index (*p* < 0.001). However, phenotypes did not differ by treatment on Session 4 (e.g., VEH STs were similar to CNO STs, etc.; Extended Data Fig. 3-1*d*). Thus, treatment groups were adequately counterbalanced within phenotype, and any changes in behavior between treatment groups thereafter would presumably be due to LH–PVT inhibition.

10.1523/ENEURO.0174-26.2026.f4-1 Figure 4-1 **Chemogenetic inhibition of the LH**–**PVT pathway does not impact food cup entries during the inter-trial interval.** Data are expressed as mean ± SEM for food cup entries during the inter-trial interval (ITI) within Pavlovian conditioned approach (PavCA). Food cup entries during the ITI period across sessions 1–7 (a) between treatment groups collapsed and (b–d) with treatment and phenotype groups expanded. On sessions 5–7, rats received either VEH or CNO prior to testing. There were no treatment effects on food cup entries during the ITI period (a) between treatment groups or (b–d) within any of the phenotypes for sessions 5–7. Download Figure 4-1, TIF file.

10.1523/ENEURO.0174-26.2026.f4-2 Figure 4-2 Prior chemogenetic inhibition of the LH–**PVT pathway does not impact the conditioned reinforcing properties of the lever-cue.** Data are expressed as mean ± SEM for (a,c) nose pokes in the inactive and active ports and (b,d) lever contacts during the conditioned reinforcement test (CRT). There are no effects of treatment in the CRT. (a,c) Rats nose poke in the active port more than the inactive port. (c) STs have more active than inactive nose pokes; and they have more active nose pokes than IRs and GTs. (d) STs have more lever contacts than IRs and GTs. Straight line indicates a significant effect of port. Brackets indicate significant differences between phenotypes or ports, **p < 0.01 and ***p < 0.001. Download Figure 4-2, TIF file.

10.1523/ENEURO.0174-26.2026.f4-3 Figure 4-3 Clozapine-N-oxide has no effects on Pavlovian Conditioned Approach (PavCA) behavior in the absence of DREADD receptors. Data are expressed as mean ± SEM for rats that did not receive surgery for DREADD expression during Pavlovian conditioned approach (PavCA). (a–c) Sign-tracking and (d–f) goal-tracking behaviors across sessions 1–7 shown with treatment groups (black lines) collapsed in the foreground and with phenotype/treatment groups expanded in background (muted color). (a–c) Lever- and (d–f) food cup-directed behaviors for (a,d) number of contacts/entries, (b,e) probability to approach, or (c,f) latency to approach. Treatment had no effect on sign- or goal-tracking behaviors. Download Figure 4-3, TIF file.

10.1523/ENEURO.0174-26.2026.f4-4 Figure 4-4 Clozapine-N-oxide has no effects on the conditioned reinforcing properties of the lever in the absence of DREADD expression. Data are expressed as mean ± SEM for rats that did not receive surgery for DREADD expression during (a–d) a conditioned reinforcement test (CRT). Behavior during the CRT is shown as (a–b) treatment groups collapsed and (c–d) treatment and phenotype groups expanded. (a,c) Nose pokes in the inactive and active ports and (b,d) lever contacts. There were no significant effects of treatment on behavior during the CRT. (a,c) Rats nose poke in the active port more than the inactive port. (c) STs have more active than inactive nose pokes. (d) STs have more lever contacts than IRs and GTs. Straight line indicates a significant effect of port. Brackets indicate significant differences between phenotypes or ports, **p < 0.01 and ***p < 0.001. Download Figure 4-4, TIF file.

10.1523/ENEURO.0174-26.2026.f4-5 Figure 4-5 Statistical analyses for the effects of treatment and phenotype on food cup entries during the inter-trial interval. Data are from linear mixed effects model analyses for (a) sessions 1–4 and (b) sessions 5–7; (c) shows follow-up treatment comparisons within phenotype for the significant session × treatment × phenotype interaction. For (a,b), the left panel compares rats by treatment, collapsed across phenotype, and the right panel compares rats with treatment and phenotype as variables. Significant effects and interactions are bolded. These data correspond to data shown in Figure 4-1. Download Figure 4-5, DOCX file.

10.1523/ENEURO.0174-26.2026.f4-6 Figure 4-6 **Statistical analyses for behavior during the Conditioned Reinforcement Test (CRT).** Data from a mixed ANOVA comparing (left column) nose pokes into each port and a two-way ANOVA comparing lever contacts during the conditioned reinforcement test. Analysis was conducted (a) between treatment groups and (b) with treatment and phenotype as variables. Significant effects and interactions are bolded. These data correspond to the data shown in Figure 4-2. Download Figure 4-6, DOCX file.

10.1523/ENEURO.0174-26.2026.f4-7 Figure 4-7 **Statistical analyses for sign- and goal-tracking behaviors in the absence of DREADD.** Data from linear mixed effects model analyses assessing the effect of treatment in rats that did not have surgery for DREADD expression. Results are shown for (a,d) sessions 1–4 and (b,e) sessions 5–7. Contacts/entries, probability, and latency are represented for (a–b) sign-tracking behaviors and (d–e) goal-tracking behaviors. Significant effects and interactions are bolded. These data correspond to that shown in Figure 4-3. Download Figure 4-7, DOCX file.

10.1523/ENEURO.0174-26.2026.f4-8 Figure 4-8 Statistical analyses for the effects of treatment and phenotype on sign-tracking behaviors in the absence of DREADD. Data from linear mixed effects model analyses assessing the effect of treatment in rats that did not have surgery for DREADD expression. Results are shown for (a) sessions 1–4 and (b) sessions 5–7, comparing rats by treatment and phenotype. Contacts, probability, and latency are represented for sign-tracking behaviors. Significant effects and interactions are bolded. These data correspond to that shown in Figure 4-3. Download Figure 4-8, DOCX file.

10.1523/ENEURO.0174-26.2026.f4-9 Figure 4-9 **Statistical analyses for the effects of treatment and phenotype on goal-tracking behaviors in the absence of DREADD.** Data from linear mixed effects model analyses in rats that did not have surgery for DREADD expression. Data are shown for (a) sessions 1–4 and (b) sessions 5–7, comparing rats by treatment and phenotype, and (c) shows follow-up analyses within each phenotype for the significant session × treatment × phenotype interaction for food cup entries. Entries, probability, and latency are represented for goal-tracking behaviors. Significant effects and interactions are bolded. These data correspond to that shown in Figure 4-3. Download Figure 4-9, DOCX file.

10.1523/ENEURO.0174-26.2026.f4-10 Figure 4-10 **Statistical analyses for behavior during the Conditioned Reinforcement Test (CRT) in the absence of DREADD.** Data from a mixed ANOVA comparing (left column) nose pokes into each port and a two-way ANOVA comparing lever contacts during the conditioned reinforcement test. Analysis was conducted between (a) treatment groups and (b) with treatment and phenotype as variables. Significant effects and interactions are bolded. These data correspond to that shown in Figure 4-4. Download Figure 4-10, DOCX file.

**Table 2.** Statistical analyses for effects of treatment and phenotype on sign-tracking behaviors

	Sessions 1–4: sign-tracking behaviors								
	Lever contacts			Probability lever			Latency lever		
	DF	*F*	*p*	DF	*F*	*p*	DF	*F*	*p*
Session	3,137.409	32.727	**<0.001**	3,73.415	25.881	**<0.001**	3,59.093	29.867	**<0.001**
Treatment	1,50.676	0.894	0.349	1,50.507	0.961	0.331	1,48.867	1.043	0.312
Phenotype	2,50.676	27.831	**<0.001**	2,50.507	40.353	**<0.001**	2,48.867	34.499	**<0.001**
Session × treatment	3,137.409	0.152	0.928	3,73.415	0.774	0.512	3,59.093	0.813	0.492
Session × phenotype	6,137.409	18.003	**<0.001**	6,73.415	13.343	**<0.001**	6,59.093	16.300	**<0.001**
Treatment * phenotype	2,50.676	1.040	0.361	2,50.507	0.923	0.404	2,48.867	1.127	0.332
Session × treatment × phenotype	6,137.409	0.237	0.964	6,73.415	2.135	0.059	6,59.093	1.053	0.401
	Sessions 5–7: sign-tracking behaviors								
	Lever contacts			Probability lever			Latency lever		
	DF	*F*	*p*	DF	*F*	*p*	DF	*F*	*p*
Session	2,70.114	0.364	0.696	2,95.565	1.123	0.329	2,72.946	0.551	0.579
Treatment	1,48.057	0.009	0.923	1,49.042	0.036	0.850	1,48.227	0.686	0.412
Phenotype	2,48.057	22.509	**<0.001**	2,49.042	26.715	**<0.001**	2,48.227	30.622	**<0.001**
Session × treatment	2,70.114	0.254	0.776	2,95.565	0.173	0.841	2,72.946	0.058	0.944
Session × phenotype	4,70.114	1.636	0.175	4,95.565	1.160	0.333	4,72.946	1.710	0.157
Treatment * phenotype	2,48.057	0.038	0.962	2,49.042	0.019	0.981	2,48.227	0.091	0.914
Session × treatment × phenotype	4,70.114	0.551	0.699	4,95.565	0.890	0.473	4,72.946	0.274	0.894
	Pretest versus test: sign-tracking behaviors in IRs								
	Lever contacts			Probability lever			Latency lever		
	DF	*F*	*p*	DF	*F*	*p*	DF	*F*	*p*
Session	1,16	3.650	0.074	1,16	2.767	0.116	1,16	3.829	0.068
Treatment	1,16	0.000	0.984	1,16	0.038	0.847	1,16	0.056	0.816
Session × treatment	1,16	0.400	0.536	1,16	0.008	0.928	1,16	0.321	0.579

Data from LMM analyses for sessions 1–4 and sessions 5–7, with comparisons by treatment and phenotype. A two-way repeated-measures ANOVA assessing the effect of treatment within IRs during pretest versus test sessions. Contacts, probability, and latency are represented for sign-tracking behaviors. Significant effects and interactions are bolded. These data correspond to that shown in Figure 4.

**Table 3.** Statistical analyses for effects of treatment and phenotype on goal-tracking behaviors

	Sessions 1–4: goal-tracking behaviors								
	Food cup entries			Probability food cup			Latency food cup		
	DF	*F*	*p*	DF	*F*	*p*	DF	*F*	*p*
Session	3,76.837	7.035	**<0.001**	3,95.188	18.529	**<0.001**	3,86.212	17.968	**<0.001**
Treatment	1,51.163	0.278	0.601	1,50.286	0.022	0.883	1,51.460	0.001	0.972
Phenotype	2,51.163	4.666	**0.014**	2,50.286	5.562	**0.007**	2,51.460	7.078	**0.002**
Session × treatment	3,76.837	0.284	0.837	3,95.188	0.260	0.854	3,86.212	0.068	0.977
Session × phenotype	6,76.837	11.583	**<0.001**	6,95.188	19.421	**<0.001**	6,86.212	17.423	**<0.001**
Treatment × phenotype	2,51.163	0.409	0.666	2,50.286	0.056	0.946	2,51.460	0.303	0.740
Session × treatment × phenotype	6,76.837	0.665	0.678	6,95.188	1.562	0.167	6,86.212	0.881	0.512
	Sessions 5–7: goal-tracking behaviors								
	Food cup entries			Probability food cup			Latency food cup		
	DF	*F*	*p*	DF	*F*	*p*	DF	*F*	*p*
Session	2,64.226	0.203	0.817	2,95.255	0.812	0.447	2,69.999	1.615	0.206
Treatment	1,48.429	4.349	**0.042**	1,48.154	7.561	**0.008**	2,48.804	3.945	0.053
Phenotype	2,48.436	32.846	**<0.001**	2,48.161	57.539	**<0.001**	2,48.811	56.982	**<0.001**
Session × treatment	2,64.226	0.563	0.572	2,95.255	0.579	0.563	2,69.999	0.083	0.920
Session × phenotype	4,64.283	0.451	0.771	4,95.255	0.097	0.983	4,70.005	0.234	0.918
Treatment × phenotype	2,48.436	1.426	0.250	2,48.161	4.886	**0.012**	2,48.811	2.655	0.080
Session × treatment × phenotype	4,64.283	1.742	0.152	4,95.255	1.752	0.145	4,70.005	1.938	0.114
	Pretest versus test: goal-tracking behaviors in IRs								
	Food cup entries			Probability food cup			Latency food cup		
	DF	*F*	*p*	DF	*F*	*p*	DF	*F*	*p*
Session	1,16	0.035	0.853	1,16	0.016	0.901	1,16	0.165	0.690
Treatment	1,16	5.560	**0.031**	1,16	5.149	**0.037**	1,16	2.998	0.103
Session × treatment	1,16	4.448	0.051	1,16	8.311	**0.011**	1,16	4.105	0.060

Data from LMM analyses for sessions 1–4 and sessions 5–7, with comparisons by treatment and phenotype. A two-way repeated-measures ANOVA assessing the effect of treatment within IRs during pretest versus test sessions. Entries, probability, and latency are represented for goal-tracking behaviors. Significant effects and interactions are bolded. These data correspond to that shown in Figure 4.

### Effects of inhibiting the LH–PVT pathway on PavCA behavior (test)

#### Inhibition of the LH–PVT pathway has no impact on sign-tracking

Chemogenetic inhibition of the LH–PVT pathway via systemic CNO administration had no effect on the expression of sign-tracking behaviors (Tables 1, 2). There were no significant differences in the number of lever-cue contacts (Figs. 3*a*, 4*a*), probability to approach the lever-cue (Figs. 3*c*, 4*c*), or latency to approach the lever-cue (Figs. 3*e*, 4*e*) between VEH- and CNO-treated rats on Sessions 5–7. This was true when comparing treatment groups collapsed across phenotypes (Table 1; Fig. 3*a*,*c*,*e*) and when phenotype was included as a variable (Table 2; Fig. 4*a*,*c*,*e*). As expected, sign-tracking behavior changed across sessions, and there was a significant effect of phenotype for each lever-directed measure (Table 2). Post hoc comparisons revealed that STs had significantly more lever-cue contacts (*p* < 0.001; Fig. 4*a*), a higher probability to approach the lever-cue (*p* < 0.001; Fig. 4*c*), and a faster latency to approach the lever-cue (*p* < 0.001; Fig. 4*e*) relative to IRs and GTs on Sessions 5–7. Thus, LH–PVT pathway inhibition via DREADD activation had no effect on sign-tracking behavior.

To further assess differences in behavior as a function of LH–PVT pathway inhibition, we directly compared sign-tracking behaviors on Session 4 (pretest) to the average of Sessions 5–7 (test; Tables 1, 2). There was a significant effect of session for all lever-directed behaviors (Table 1; Fig. 3*b*,*d*,*f*). Post hoc comparisons revealed that rats made more lever-cue contacts (*p* = 0.008; Fig. 3*b*), had a higher probability to approach the lever-cue (*p* = 0.005; Fig. 3*d*), and had a faster latency to approach the lever-cue (*p* = 0.002; Fig. 3*f*) during test sessions relative to pretest. This continued change in responding is expected with subsequent training sessions (Meyer et al., 2012). Consistent with these findings, sign-tracking behavior was not altered due to LH–PVT inhibition when directly comparing pretest to test sessions within phenotypes (Table 2, Fig. 4*b*,*d*,*f*; data not shown for ST or GT).

#### Inhibition of the LH–PVT pathway decreases goal-tracking behavior

Chemogenetic inhibition of the LH–PVT pathway decreased goal-tracking behaviors, and this reduction was phenotype-dependent. When collapsed across phenotype, there was a significant effect of treatment for goal-tracking behavior on Sessions 5–7 (Table 1). Relative to VEH-treated rats, CNO-treated rats had decreased food cup entries when the lever-cue was presented (effect of treatment, *F*_(1,52.137)_ = 5.147; *p* = 0.027; Fig. 3*g*), decreased probability to approach the food cup (effect of treatment, *F*_(1,52.025)_ = 5.119; *p* = 0.028; Fig. 3*i*), and a trend for an increased latency to approach the food cup (effect of treatment, *F*_(1,52.012)_ = 3.655; *p* = 0.061; Fig. 3*k*). Thus, inhibition of the LH–PVT pathway decreased the expression of goal-tracking behavior. Together with the lack of effect on sign-tracking, these data suggest that LH–PVT inhibition selectively reduced food cup-directed conditioned responding.

When phenotype was considered as a variable, there was a significant effect of phenotype (*p* < 0.001) and treatment (*p* ≤ 0.05) for all measures of goal-tracking behavior over Sessions 5–7 (Table 3) and a significant phenotype × treatment interaction for the probability to approach the food cup (*F*_(2,48.436)_ = 4.886; *p* = 0.012). This interaction indicated that the treatment effect was not evenly distributed across phenotypes. As expected, GTs showed more robust responding directed toward the food cup relative to STs, and this was independent of treatment (Table 3; Fig. 4*g*,*i*,*k*). As shown in Figure 4*i*, however, VEH-treated IR rats showed a pattern of food cup approach more similar to GTs, whereas CNO-treated IR rats showed reduced food cup approach and a pattern more similar to STs by the end of the testing phase. In agreement, post hoc comparisons revealed that CNO-treated IRs had a significantly reduced probability to approach the food cup relative to VEH-treated IRs (*p* < 0.001; Cohen's *d* = 1.53). Furthermore, within treatment groups, VEH-treated IRs had a significantly higher probability to approach the food cup relative to VEH-treated STs (*p* < 0.001; Cohen's *d* = 2.48), but not VEH-treated GTs (*p* = 0.372), whereas CNO-treated IRs had a significantly lower probability to approach the food cup relative to CNO-treated GTs (*p* < 0.001; Cohen's *d* = 2.65), but not CNO-treated STs (*p* = 0.063). Taken together, inhibition of the LH–PVT pathway significantly and selectively decreased the probability to goal-track in IR rats.

We also compared Session 4 (pretest) to the average of Sessions 5–7 (test) for goal-tracking behaviors in VEH- and CNO-treated rats (Table 1; Fig. 3*h*,*j*,*l*). As shown in Figure 3*j*, there was a significant session × treatment interaction for probability to approach the food cup when the lever-cue was presented (*F*_(1,52)_ = 10.532; *p* = 0.002). Post hoc comparisons revealed that CNO-treated rats had a significantly higher probability to approach the food cup on the pretest session relative to the test session (*p* = 0.005; Cohen's *d* = 0.35). Additionally, on the test session, CNO-treated rats had a significantly lower probability to approach the food cup relative to VEH-treated rats (*p* = 0.028; Cohen's *d* = 0.15). Thus, the pretest–test comparison supported the Session 5–7 analysis, showing that LH–PVT inhibition reduced the probability of food cup approach during cue presentation.

We further compared goal-tracking behaviors between pretest and test sessions in IRs (Table 3). As shown in Figure 4*j*, there was a significant session × treatment interaction (*F*_(1,16)_ = 8.311; *p* = 0.011) for probability to approach the food cup. Post hoc comparisons revealed that CNO-treated IRs had a significantly higher probability to approach the food cup on the pretest session relative to the test session (*p* = 0.042; Cohen's *d* = 0.68). Additionally, on the test session, CNO-treated IR rats had a significantly lower probability to approach the food cup relative to VEH-treated IR rats (*p* = 0.006; Cohen's *d* = 0.36). Taken together, these data support those above, demonstrating that LH–PVT inhibition attenuates the probability to goal-track and does so selectively in IR rats.

#### Inhibition of the LH–PVT pathway does not alter food cup entries during the ITI

To determine whether LH–PVT inhibition reduced food cup-directed behavior more broadly, we analyzed food cup entries during the ITI, when the lever-cue was not present. There was an overall decrease in ITI food cup entries across Sessions 1–4 (effect of session, *F*_(3,92.361)_ = 5.794; *p* = 0.001) and 5–7 (effect of session, *F*_(2,97.023)_ = 15.236; *p* < 0.001; Extended Data Figs. 4-1*a*, 4-5*a,b*), as expected with learning of the cue–reward relationship. There was no effect of treatment on ITI food cup entries during Sessions 5–7 when rats were analyzed with phenotypes collapsed (Extended Data Figs. 4-1*a*, 4-5*b*), and this was also true when phenotype was included as a variable (Extended Data Figs. 4-1*b–d*, 4-5*b*). Although there was a significant session × treatment × phenotype interaction for food cup entries during Sessions 5–7 (*F*_(4,48.007)_ = 3.251; *p* = 0.019), follow-up comparisons showed no significant VEH–CNO differences within STs, IRs, or GTs (Extended Data Fig. 4-5*c*). Since food cup entries during the ITI were not affected by treatment either in the total population or in IRs, LH–PVT inhibition appears to selectively reduce cue-evoked goal-tracking behavior without a general reduction in food cup entries. In agreement, all rats consumed all of the food pellets that were delivered during PavCA sessions.

#### Inhibition of the LH–PVT pathway during PavCA does not affect the conditioned reinforcing properties of the lever-cue

Prior treatment with CNO during PavCA did not affect performance on the subsequent CRT, which was conducted in the absence of acute VEH or CNO treatment. Thus, LH–PVT inhibition during PavCA did not alter the conditioned reinforcing properties of the lever-cue under these conditions (Extended Data Figs. 4-2, 4-6).

#### CNO in the absence of DREADD does not impact behavior

The purpose of Experiment 2 was to confirm that the effects reported above were DREADD-mediated; therefore, we assessed the effects of CNO (5 mg/kg, i.p.) in the absence of DREADDs in the brain. CNO-treated rats were no different from VEH-treated rats in sign-tracking or goal-tracking behaviors during PavCA Sessions 1–4 or Sessions 5–7 (Extended Data Figs. 4-3, 4-7), and the same was true when phenotype was considered as a variable (Extended Data Figs. 4-3, 4-9). Although there was a significant session × treatment × phenotype interaction for food cup entries during Sessions 5–7 (*F*_(4,110.272)_ = 3.112; *p* = 0.018; Extended Data Fig. 4-9*b*), follow-up analyses within phenotype did not reveal significant session × treatment interactions in STs, IRs, or GTs (Extended Data Fig. 4-9*c*). Thus, CNO did not produce a treatment-dependent change in food cup entries in rats without DREADD expression. Furthermore, prior VEH or CNO treatment did not alter the conditioned reinforcing properties of the lever-cue (Extended Data Figs. 4-4, 4-10). Thus, while STs, IRs, and GTs showed the expected behavioral patterns, VEH- and CNO-treated rats did not differ within any phenotype in the absence of DREADD. Together, the CNO-elicited effects reported above were not attributable to CNO alone.

## Discussion

We examined the role of the LH–PVT pathway in the expression of CR in a PavCA paradigm. Chemogenetic inhibition of this pathway decreased goal-tracking behavior, with effects confined to IRs when the PavCA phenotype was considered. Thus, LH–PVT signaling supports goal-tracking behavior in a phenotype-dependent manner. Notably, all rewards were consumed during PavCA sessions, and LH–PVT inhibition did not impact food cup entries during the ITI. Thus, the reduction in goal-tracking appears specific to cue-evoked food cup-directed responding rather than reflecting disrupted reward consumption or a general reduction in food cup-directed behavior.

The selective impact of LH–PVT inhibition on IRs is consistent with the idea that once a prepotent CR is established, it can be difficult to disrupt, particularly in animals at the extremes of the population (Flagel et al., 2009; Campus et al., 2019). IRs display flexibility in their responding, vacillating between cue- and food cup-directed behaviors, and may therefore be especially sensitive to manipulations that bias response selection. This interpretation aligns with evidence that the PVT integrates cortical and hypothalamic signals to shape cue–reward processing and reward seeking (Otis et al., 2019) and may arbitrate motivational competition when multiple responses are available (Choi et al., 2019; McNally, 2021). The IR-specific effect was most apparent in the probability of food cup approach, such that LH–PVT inhibition reduced the likelihood that IR rats initiated a goal-tracking response during cue presentation. Measures of response vigor and approach latency were less affected, suggesting an effect on response selection rather than general performance. Because most prior work has emphasized ST and GT extremes (Flagel et al., 2007; Robinson and Flagel, 2009; Campus et al., 2019; Haight et al., 2020), the circuit mechanisms supporting IR behavior remain poorly characterized. The current findings identify the LH–PVT pathway as a potential leverage point for the flexible cue-motivated responding seen in IRs, a behavioral pattern that may better capture population-level variability.

The absence of an effect on sign-tracking may appear to diverge from work emphasizing bottom–up contributions to incentive salience attribution in STs. Prior studies implicate hypothalamic–thalamic–striatal circuitry in incentive motivational processes (Flagel et al., 2011a; Haight et al., 2020), making it reasonable to predict that suppressing LH–PVT signaling would attenuate sign-tracking. However, our data indicate that broad LH–PVT inhibition is not sufficient to disrupt the expression of sign-tracking behavior. One possibility is that sign-tracking depends on specific components of the LH–PVT projection, particularly orexinergic transmission, rather than global pathway activity. Consistent with this, orexin receptor antagonism in the PVT reduces the incentive value of a food-paired cue in STs (Haight et al., 2020). Additionally, LH–PVT signaling may be more critical during acquisition than expression of CR. Supporting this possibility, excitotoxic lesions of the LH performed before learning reduced subsequent sign-tracking behavior (Haight et al., 2020). Moreover, pPVT activity gates associative learning during acquisition but not expression (Zhu et al., 2018). In head-fixed mice, optogenetic silencing of the PVT during cue (CS) presentation or reward (US) consumption weakened CS–US learning, reflected by reduced anticipatory licking, but only when delivered during acquisition (Zhu et al., 2018). Together, these findings motivate targeted tests of cell-type- and phase-specific mechanisms (acquisition vs expression) within LH–PVT circuitry.

A key limitation of the present approach is that the viral manipulation was not cell-type specific and likely engaged multiple LH–PVT systems. LH–PVT projections are heterogeneous, including dense orexinergic, GABAergic, and glutamatergic inputs (Kirouac et al., 2005; Li and Kirouac, 2012; Jennings et al., 2015; Otis et al., 2019), and the PVT itself contains molecularly distinct neuronal subtypes (Gao et al., 2023). Accordingly, distinct LH–PVT populations likely contribute to reward-related behavior via different mechanisms. Orexinergic signaling in the PVT promotes appetitive motivation (Li et al., 2009; Stratford and Wirtshafter, 2013; Barson et al., 2015), including sign-tracking (Haight et al., 2020). However, orexinergic neurons do not signal exclusively through orexin; they can also contain and release dynorphin (Chou et al., 2001; Li and van den Pol, 2006). Thus, behavioral effects of broad LH–PVT inhibition may also involve dynorphin signaling. In addition, melanin-concentrating hormone (MCH)-containing neurons have been broadly implicated in motivated behaviors (for review, see Diniz and Bittencourt, 2017), including consummatory processes and appetitive food-seeking in response to discrete and contextual food-predictive cues (Subramanian et al., 2023). Hypothalamic peptidergic projections to the PVT that have been implicated in feeding and arousal also include MCH-containing inputs (Lee et al., 2015). Therefore, although prior work supports the role of orexinergic signaling within the LH–PVT pathway (Haight et al., 2020), the current manipulation may have also affected additional peptidergic signaling, including dynorphin and MCH.

In parallel, LH–PVT GABAergic projections are substantial (Otis et al., 2019), and their stimulation elicits consummatory and reward seeking behaviors (Wu et al., 2015; Zhang and van den Pol, 2017), whereas optogenetic inhibition of LH GABA neurons after learning reduces motivation to seek reward without altering consumption (Sharpe et al., 2017). Moreover, distinct LH GABAergic subpopulations appear to differentially track reward seeking versus consumption (Jennings et al., 2015). This is relevant to our findings, which show reduced goal-tracking without changes in reward consumption, suggesting that LH–PVT inhibition disrupted cue-evoked approach rather than primary reinforcement or consummatory processes. However, because feeding was not assessed outside of PavCA, the absence of a consumption effect here should not be taken as evidence that LH–PVT signaling is uninvolved in feeding more broadly (Hoebel and Teitelbaum, 1962; Margules and Olds, 1962). Consistent with broader feeding roles, the LH is functionally heterogeneous and can support both food intake and self-stimulation depending on stimulation site and experience (Urstadt and Berridge, 2020). Furthermore, the PVT is embedded in feeding-related circuitry via connections to the NAc shell (Stratford, 2005, 2007) and hypothalamus (Chen and Su, 1990; Moga et al., 1995; Stratford, 2005, 2007), and activation of GABA_A_ receptors in the PVT dose-dependently increases food intake in sated rats when food is freely available (Stratford and Wirtshafter, 2013). Methodological differences (e.g., systemic CNO inhibition of LH–PVT neurons during PavCA vs local manipulation of PVT GABA_A_ signaling during a feeding test) likely also contribute to divergent outcomes across studies. This point is further supported by evidence that orexin receptor 1–expressing PVT neurons are activated in response to food-paired cues (Choi et al., 2010) and GABAergic LH–PVT inputs onto glutamatergic PVT–NAc neurons have been shown to mediate consummatory behavior, such as licking (Otis et al., 2019). These distinctions may help explain why LH–PVT manipulations affect reward seeking and consummatory behavior differently across paradigms and under conditions that vary satiety state and food availability.

Anatomical specificity is also important to consider, as mCherry-positive cell bodies were consistently observed in the ZI, an LH-adjacent region that is difficult to avoid with the viral targeting approach we utilized. The ZI sends dense GABAergic projections to the PVT (Kirouac et al., 2006; Ye and Zhang, 2021; Yang et al., 2022; Aquino-Miranda et al., 2024), and this pathway has been implicated in food-seeking and consumption, with stimulation evoking binge-like eating (Zhang and van den Pol, 2017; Ye et al., 2023). The ZI also sends dopaminergic projections to the PVT, which have been proposed to encode motivational vigor during food-seeking (Ye et al., 2023). Thus, viral expression in the broader LH/ZI region may have affected multiple PVT-projecting populations, including peptidergic populations relevant to the MCH-related caveat noted above. Although the current study cannot fully dissociate LH–PVT from adjacent ZI–PVT contributions, the data described above argue against a general disruption of feeding or appetitive behavior. Instead, the present findings suggest that inhibition of PVT-projecting neurons within the LH/ZI region selectively alter conditioned food cup-directed responding during cue presentation. Future studies using more spatially restricted and cell-type-specific approaches will be needed to dissociate the respective contributions of LH–PVT and ZI–PVT projections to cue-motivated behavior and to determine the relative involvement of neurochemically defined populations, including orexinergic, dynorphin-containing, MCH-containing, dopaminergic, GABAergic, and glutamatergic neurons.

To date, PVT-focused studies within the ST/GT model have largely targeted the entire PVT rather than isolating anterior or posterior subregions (Haight et al., 2015, 2017; Kuhn et al., 2018, 2022; Campus et al., 2019). However, the aPVT and pPVT have distinct functions. Generally, the aPVT appears to regulate reward seeking and arousal (Kolaj et al., 2012; Do-Monte et al., 2017; Cheng et al., 2018; Gao et al., 2020), whereas the pPVT is largely involved in stress and anxiety (Bhatnagar and Dallman, 1999; Li et al., 2010a,b; Barson and Leibowitz, 2015). Because we targeted the entire rostrocaudal extent of the PVT, it is difficult to determine whether the observed effects arise primarily from aPVT, pPVT, or their interaction. Further supporting this point, recent work shows that parallel PVT projections to the NAc can dissociably encode features of goal-oriented behavior, including the execution and termination of goal-directed actions (Beas et al., 2024). This is especially relevant given that LH inputs, including orexinergic projections, may differentially engage anterior and pPVT subregions, which could have distinct consequences for cue-motivated behavior. Selective targeting of the aPVT and pPVT would clarify whether LH–PVT projections regulate distinct features of PavCA behavior through subregion-specific PVT circuits. Because orexinergic LH–PVT projections have been implicated in incentive salience attribution (Haight et al., 2017, 2020), orexinergic LH–aPVT projections may be a particularly important target for future studies in this model.

Together with our findings, recent work supports viewing the PVT and LH–PVT pathway as a collection of specialized, state-dependent circuits rather than a single functional unit. This view is consistent with the early framework proposed by Kelley and colleagues, in which the PVT was positioned as a critical node in a hypothalamic–thalamic–striatal circuit that integrates state-related signals to guide motivated behaviors (Kelley et al., 2005). More recent work suggests that PVT contributions to motivated behavior are best understood through projection-defined and state-dependent circuit mechanisms rather than through whole-region manipulations alone (Beas et al., 2024). Consistent with this view, distinct PVT–NAc circuits may differentially encode valence and salience (Rivera-Irizarry et al., 2023), while PVT dopamine D2R+ neurons integrate hunger, thirst, and cue-related information to guide motivated behavior (Machen et al., 2026). Collectively, these findings suggest that the behavioral effects observed here may depend on the specific LH inputs engaged, the PVT subregion and output pathway recruited, and the motivational state of the animal. Whether LH–PVT inhibition regulates PavCA behavior similarly across internal states such as satiety, food restriction, stress, or altered arousal remains an open question.

Although inclusion of both sexes was intended for this work, we were unable to add females to the study due to the COVID-19 pandemic and subsequent resource constraints. Thus, the current findings are limited to male rats. It remains unclear whether LH–PVT inhibition would produce similar effects in females. This limitation warrants consideration, as sex differences have been reported in PavCA behavior (Hughson et al., 2019; Stringfield et al., 2019) and in neural systems that regulate reward seeking and motivated behavior (Becker and Koob, 2016; Becker et al., 2017). However, the direction of these effects is difficult to predict, as sex differences in PavCA behavior appear to depend on experimental conditions, vendor/strain, behavioral measure, and exposure history (Bien and Smith, 2023; Turfe et al., 2024). Future work should include both males and females to determine whether the LH–PVT pathway regulates goal-tracking behavior and behavioral flexibility similarly across sex.

Sample sizes in the current study were limited by uneven phenotype distributions across vendor/barrier cohorts and by stringent anatomical inclusion criteria for DREADD expression. This resulted in uneven group sizes, particularly within phenotype-specific comparisons. Although this limitation may reduce sensitivity to detect smaller effects, the primary IR effect was large, particularly for probability to approach the food cup (Cohen's *d* = 1.53). In contrast, variability within the ST and GT treatment groups was low, suggesting that the absence of treatment effects in these phenotypes was not simply due to increased variance in behavioral responding.

There were no effects of CNO on behavior during PavCA or CRT in rats that did not receive viral infusions for DREADD expression. While these findings support the interpretation that the behavioral effects observed here were not attributable to CNO alone (Gomez et al., 2017; Goutaudier et al., 2019), the current study did not include a control-virus or control-DREADD group. Thus, we cannot fully exclude potential viral- or DREADD-induced effects independent of hM4Di-mediated inhibition of the LH–PVT pathway. Future studies would benefit from including fluorophore-only or control-DREADD groups to more fully isolate the effects of pathway-specific inhibition.

In the current study, we targeted afferents from the LH to the PVT to elucidate the role of this pathway in phenotypically distinct cue-motivated behaviors. CNO administration in non-DREADD rats had no off-target effects in PavCA or CRT, supporting the interpretation that the behavioral effects observed here were DREADD-mediated. Together, these data indicate that LH–PVT pathway inhibition reduces reward-directed conditioned responding in behaviorally flexible PavCA phenotypes without broadly disrupting sign-tracking, ITI food cup entries, or reward consumption within the testing context. Future studies should combine cell-type-specific manipulations within the LH with subregion-specific targeting of the PVT to dissociate contributions of orexinergic and non-orexinergic signaling. Collectively, these findings refine the role of the LH–PVT pathway in cue-motivated behavior and suggest that this circuit is especially relevant when reward-directed CR are flexible rather than strongly biased toward the cue or reward location.

## Synthesis

Reviewing Editor: Sam Golden, The University of Washington Decisions are customarily a result of the Reviewing Editor and the peer reviewers coming together and discussing their recommendations until a consensus is reached. When revisions are invited, a fact-based synthesis statement explaining their decision and outlining what is needed to prepare a revision will be listed below. The following reviewer(s) agreed to reveal their identity: Gilbert Kirouac, Jessica Barson.

Dear Dr. Flagel,

Thank you for submitting your manuscript to eNeuro, it has been reviewed by two experts in the field. Both reviewers were enthusiastic about the quality and impact of the manuscript and provided only minor critiques that can be Editorially Reviewed. Below please find a synthesis of these comments:

Reviewer 1:

Congratulations on a extremely well executed series of experiments and written manuscript. The authors should be commended for their efforts at incorporating their findings within what is known about the PVT and its control of behavior.

Minor issue:

The density of DREADDs expression in the zona incerta compared to the LH is rather surprising considering this has not been consistently reported in the tracing literature. Maybe a few related comments could be added.

Reviewer 2:

This study examines male rats for their responses to a Pavlovian conditioned approach paradigm, and the influence of the neuronal pathway from the lateral hypothalamus (LH) to the paraventricular nucleus of the thalamus (PVT) on these responses. From the Pavlovian conditioned approach paradigm, rats can be classified as sign-trackers (STs), goal-trackers (GTs), or intermediate responders (IRs), based on their level of approach and interaction with the cue compared to the location of reward delivery. With chemogenetic inhibition of the LH-PVT pathway, the authors find that goal-tracking but not sign-tracking can be attenuated, and that this primarily occurs in the IR population. In a control experiment, they find that injection of the DREADD actuator, CNO, does not significantly alter behavior. The authors conclude that the LH-PVT pathway is a selective contributor to reward-directed conditioned responding and a circuit substrate for behavioral flexibility.

This is a straightforward and well-written manuscript. It includes thoughtful citation of foundational works in the Introduction, a large number of subjects and appropriate statistical tests in the Methods and Results, and a reasonable consideration of most major limitations in the Discussion. I have only a few suggested changes, which I hope will strengthen the final product.

Specific Issues:

1. There seems to be a major focus in the Introduction and Discussion on how the LH sends orexinergic projections to the PVT, and this is indeed a major projection. However, it is worth discussing that these orexin projections also contain (and release) dynorphin, and there are also melanin-concentrating hormone (MCH) projections from the LH to the PVT. In fact, in the representative image in Figure 2, the pattern of DREADD labeling in the LH (and zona incerta) seems to follow the expression pattern of MCH-containing cells. I suggest that the authors consider somewhere in the text that their chemogenetic manipulation may have had effects on the release of MCH into the PVT and that this may be a driver of their observed behavioral effects.

2. In the Methods, I did not see any information about food restriction for the animals, to ensure that they consumed the food pellets in the chambers. If the animals were fed ad libitum, please (re-)state this in the section about Pavlovian conditioned approach (PavCA) training.

3. Why was session 4 alone used to characterize rats into their behavioral phenotypes? Is this the most reliable predictor? Please add some text to justify this selection.

4. Why was immunofluorescence required for obtaining representative images? Was the DAB processing not sufficient for this? Please explain.

5. Another limitation to this study is that there were no groups that received a control DREADD. I recognize that the sheer number of animals required for these experiments would make adding control DREADD groups a heavy lift, but this limitation should be addressed in the Discussion.

6. In the final paragraph, I believe the authors meant to type "testing" rather than "texting".

## Author Response

Reviewer #1:

1. The density of DREADDs expression in the zona incerta compared to the LH is rather surprising considering this has not been consistently reported in the tracing literature. Maybe a few related comments could be added.

We thank the reviewer for this comment and agree that the observed expression in the zona incerta should be further acknowledged. There have been several publications supporting anatomically defined projections from the ZI to the PVT, and these are now cited in the manuscript. We have also revised the Discussion to address the anatomical proximity of the LH and ZI, the possibility that the current viral approach engaged adjacent ZI-PVT neurons, and the possibility that non-orexinergic LH/ZI populations, including MCH-containing neurons, contributed to the observed behavioral effects. These points are now largely addressed in the following paragraph in the Discussion, with full references provided in the revised manuscript (pg. 26): "Anatomical specificity is also important to consider, as mCherry-positive cell bodies were consistently observed in the ZI, an LH-adjacent region that is difficult to avoid with the viral targeting approach we utilized. The ZI sends dense GABAergic projections to the PVT (Aquino-Miranda et al., 2024; Kirouac et al., 2006; Yang et al., 2022; Ye &Zhang, 2021), and this pathway has been implicated in food seeking and consumption, with stimulation evoking binge-like eating (Ye et al., 2023; Zhang &van den Pol, 2017). The ZI also sends dopaminergic projections to the PVT, which have been proposed to encode motivational vigor during food seeking (Ye et al., 2023). Thus, viral expression in the broader LH/ZI region may have affected multiple PVT-projecting populations, including peptidergic populations relevant to the MCH-related caveat noted above. Although the current study cannot fully dissociate LH-PVT from adjacent ZI-PVT contributions, the data described above argue against a general disruption of feeding or appetitive behavior. Instead, the present findings suggest that inhibition of PVT-projecting neurons within the LH/ZI region selectively alter conditioned food cup-directed responding during cue presentation. Future studies using more spatially restricted and cell-type-specific approaches will be needed to dissociate the respective contributions of LH-PVT and ZI-PVT projections to cue-motivated behavior and to determine the relative involvement of neurochemically defined populations, including orexinergic, dynorphin-containing, MCH-containing, dopaminergic, GABAergic, and glutamatergic neurons." Reviewer #2:

1. There seems to be a major focus in the Introduction and Discussion on how the LH sends orexinergic projections to the PVT, and this is indeed a major projection. However, it is worth discussing that these orexin projections also contain (and release) dynorphin, and there are also melanin-concentrating hormone (MCH) projections from the LH to the PVT. In fact, in the representative image in Figure 2, the pattern of DREADD labeling in the LH (and zona incerta) seems to follow the expression pattern of MCH-containing cells. I suggest that the authors consider somewhere in the text that their chemogenetic manipulation may have had effects on the release of MCH into the PVT and that this may be a driver of their observed behavioral effects.

We appreciate this thoughtful comment and agree that our original framing emphasized orexinergic LH-PVT projections, largely because of prior research implicating orexinergic projections from the LH to the PVT in sign- and goal-tracking behaviors. While we have left this focus in the Introduction for framing, we have revised the Discussion to acknowledge that orexinergic projections may also contain and release dynorphin, and that MCH-containing neurons play a role in responding to food-predictive cues and in driving appetitive food-seeking behavior (Lee et al., 2015; Subramanian et al., 2023). As noted above, we also expanded the Discussion (pg. 26) to address the possibility that the observed DREADD expression in the LH/ZI region may have engaged non-orexinergic peptidergic signaling, including MCH, and that future cell-type-specific approaches will be necessary to dissociate the relative contributions of orexinergic, dynorphin-containing, MCH-containing, dopaminergic, GABAergic, and glutamatergic neurons. Additional edits include the following, with full references provided in the revised manuscript (pg. 24): "Orexinergic signaling in the PVT promotes appetitive motivation (Barson et al., 2015; Li et al., 2009; Stratford &Wirtshafter, 2013), including sign-tracking (Haight et al., 2020). However, orexinergic neurons do not signal exclusively through orexin; they can also contain and release dynorphin (Chou et al., 2001; Li &van den Pol, 2006). Thus, behavioral effects of broad LH-PVT inhibition may also involve dynorphin signaling. In addition, melanin-concentrating hormone (MCH)-containing neurons have been broadly implicated in motivated behaviors (for review, see Diniz &Bittencourt, 2017), including consummatory processes and appetitive food-seeking in response to discrete and contextual food-predictive cues (Subramanian et al., 2023). Hypothalamic peptidergic projections to the PVT that have been implicated in feeding and arousal also include MCH-containing inputs (Lee et al., 2015). Therefore, although prior work supports a role for orexinergic signaling within the LH-PVT pathway (Haight et al., 2020), the current manipulation may have also affected additional peptidergic signaling, including dynorphin and MCH." 2. In the Methods, I did not see any information about food restriction for the animals, to ensure that they consumed the food pellets in the chambers. If the animals were fed ad libitum, please (re-)state this in the section about Pavlovian conditioned approach (PavCA) training.

Thank you for highlighting the need for further clarification. We previously noted in the Subjects section that rats had ad libitum access to food and water throughout the study, and we have now added the following sentence to enhance clarity (pg. 9): "Rats were not food restricted and had ad libitum access to home cage chow and water throughout behavioral testing." We also kept the two sentences in the methods section that indicate that pellet consumption was tracked during both pretraining and PavCA testing, as follows (pg. 10): "There were 25 trials, and the pretraining session lasted approximately 12.5 min, during which food cup entries were recorded, and food pellet consumption was confirmed." "It was confirmed that all food pellets had been consumed following each session." 3. Why was session 4 alone used to characterize rats into their behavioral phenotypes? Is this the most reliable predictor? Please add some text to justify this selection.

Using the average of sessions 4 and 5 has been a standard practice to assess individual variability in Pavlovian conditioned approach behavior for over a decade (Meyer et al., 2012). However, prior work has shown that PavCA phenotypes are generally stable by session 4 (Campus et al., 2019). In the current experimental design, session 4 was the final acquisition session before CNO administration and therefore provided the last pre-manipulation measure of conditioned responding. Using session 4 allowed us to categorize phenotypes and counterbalance VEH and CNO treatment groups accordingly prior to DREADD activation. We have revised the text to expand this rationale (pg. 11): "Prior studies have reported that PavCA phenotypes are generally stable at this stage of training (Campus et al., 2019). Thus, we used data from session 4 for phenotype classification because it was the final acquisition session prior to CNO administration and therefore provided the last pre-manipulation assessment of conditioned responding. This allowed VEH and CNO treatment groups to be assigned across phenotypes in a counterbalanced manner." 4. Why was immunofluorescence required for obtaining representative images? Was the DAB processing not sufficient for this? Please explain.

DAB processing was used for anatomical assessment and scoring to make inclusion/exclusion decisions. Immunofluorescence was only performed on a subset of brains to obtain publication-quality representative images of mCherry expression in the LH and PVT. We have clarified this as follows: (pg. 13): "DAB-processed tissue was used for anatomical assessment, expression scoring, and inclusion/exclusion decisions." (pg. 14): "A subset of brains was processed for immunofluorescence to obtain publication-quality representative images of mCherry expression in the LH and PVT." 5. Another limitation to this study is that there were no groups that received a control DREADD. I recognize that the sheer number of animals required for these experiments would make adding control DREADD groups a heavy lift, but this limitation should be addressed in the Discussion.

We agree and have added this limitation to the Discussion. Although our no-DREADD control experiment demonstrated that CNO alone did not impact PavCA or behavior during the conditioned reinforcement test, prior work has shown that CNO can have behavioral effects independent of DREADD expression (Gomez et al., 2017; Goutaudier et al., 2019), emphasizing the importance of appropriate controls in DREADD studies. However, the current study did not include a control-virus or control-DREADD group, and therefore we cannot fully exclude potential effects related to viral expression or DREADD expression independent of hM4Di-mediated pathway inhibition. This limitation is now addressed in the Discussion as follows (pg. 29): "There were no effects of CNO on behavior during PavCA or CRT in rats that did not receive viral infusions for DREADD expression. While these findings support the interpretation that the behavioral effects observed here were not attributable to CNO alone (Gomez et al., 2017; Goutaudier et al., 2019), the current study did not include a control-virus or control-DREADD group. Thus, we cannot fully exclude potential viral- or DREADD-induced effects independent of hM4Di-mediated inhibition of the LH-PVT pathway. Future studies would benefit from including fluorophore-only or control-DREADD groups to more fully isolate the effects of pathway-specific inhibition." 6. In the final paragraph, I believe the authors meant to type "testing" rather than "texting".

Our apologies for this typographical error, and we thank the reviewer for catching it. We have corrected "texting" to "testing" (pg. 29).

References Campus, P., Covelo, I. R., Kim, Y., Parsegian, A., Kuhn, B. N., Lopez, S. A.,...Flagel, S. B. (2019). The paraventricular thalamus is a critical mediator of top-down control of cue-motivated behavior in rats. Elife, 8. https://doi.org/10.7554/eLife.49041 Gomez, J. L., Bonaventura, J., Lesniak, W., Mathews, W. B., Sysa-Shah, P., Rodriguez, L. A.,...Michaelides, M. (2017). Chemogenetics revealed: DREADD occupancy and activation via converted clozapine. Science, 357(6350), 503-507. https://doi.org/10.1126/science.aan2475 Goutaudier, R., Coizet, V., Carcenac, C., &Carnicella, S. (2019). DREADDs: The Power of the Lock, the Weakness of the Key. Favoring the Pursuit of Specific Conditions Rather than Specific Ligands. eNeuro, 6(5). https://doi.org/10.1523/ENEURO.0171-19.2019 Lee, J. S., Lee, E. Y., &Lee, H. S. (2015). Hypothalamic, feeding/arousal-related peptidergic projections to the paraventricular thalamic nucleus in the rat. Brain Res, 1598, 97-113. https://doi.org/10.1016/j.brainres.2014.12.029 Meyer, P. J., Lovic, V., Saunders, B. T., Yager, L. M., Flagel, S. B., Morrow, J. D., &Robinson, T. E. (2012). Quantifying individual variation in the propensity to attribute incentive salience to reward cues. PLoS One, 7(6), e38987. https://doi.org/10.1371/journal.pone.0038987 Subramanian, K. S., Lauer, L. T., Hayes, A. M. R., Décarie-Spain, L., McBurnett, K., Nourbash, A. C.,...Kanoski, S. E. (2023). Hypothalamic melanin-concentrating hormone neurons integrate food-motivated appetitive and consummatory processes in rats. Nat Commun, 14(1), 1755. https://doi.org/10.1038/s41467-023-37344-9
