# Supplementary material for: Chemogenetic Inhibition of Lateral Hypothalamus Inputs to the Paraventricular Thalamus Reduces Goal-Tracking in a Behaviorally Flexible Subgroup of Male Rats
Source: eNeuro. 2026 Sep 4;13(9):ENEURO.0174-26.2026. doi: 10.1523/ENEURO.0174-26.2026 (PMC13548364; doi:10.1523/ENEURO.0174-26.2026)
Supplement: Supplementary file 1 [file eneuro-13-ENEURO.0174-26.2026-s003.docx]

|  | Figure 3-2 | | | | | | |
| --- | --- | --- | --- | --- | --- | --- | --- |
|  | **Sessions 1**–**4: PavCA Score** | | | | | | |
|  | Treatment | | |  | Treatment × Phenotype | | |
|  | DF | F | p |  | DF | *F* | *p* |
| Session | 3,73.871 | 3.164 | **.029** |  | 3,65.959 | 3.614 | **.018** |
| Treatment | 1,54.052 | 0.121 | .729 |  | 1,50.528 | 0.581 | .450 |
| Phenotype | - | - | - |  | 2,50.528 | 42.631 | **<.001** |
| Session×Treatment | 3,73.871 | 1.009 | .394 |  | 3,65.959 | 0.508 | .678 |
| Session×Phenotype | - | - | - |  | 6,65.959 | 20.242 | **<.001** |
| Treatment×Phenotype | - | - | - |  | 2,50.528 | 1.484 | .236 |
| Session×Treatment×Phenotype | - | - | - |  | 6,65.959 | 1.057 | .397 |

***Figure 3-2* Statistical analyses for PavCA Score across sessions 1**–**4.**
