# Supplementary material for: Chemogenetic Inhibition of Lateral Hypothalamus Inputs to the Paraventricular Thalamus Reduces Goal-Tracking in a Behaviorally Flexible Subgroup of Male Rats
Source: eNeuro. 2026 Sep 4;13(9):ENEURO.0174-26.2026. doi: 10.1523/ENEURO.0174-26.2026 (PMC13548364; doi:10.1523/ENEURO.0174-26.2026)
Supplement: Supplementary file 2 [file eneuro-13-ENEURO.0174-26.2026-s008.docx]

|  | Figure 4-5 | | | | | | | | | | | |
| --- | --- | --- | --- | --- | --- | --- | --- | --- | --- | --- | --- | --- |
|  | **a. Sessions 1**–**4: Food cup entries during the inter-trial interval** | | | | | | | | | | | |
|  | Treatment | | | |  | | | | Treatment × Phenotype | | | |
|  | DF | *F* | *p* | | |  |  |  | | DF | *F* | *p* |
| Session | 3,92.361 | 5.794 | **.001** | | |  |  |  | | 3,121.695 | 7.429 | **<.001** |
| Treatment | 1,58.829 | 1.356 | .249 | | |  |  |  | | 1,50.293 | 1.514 | .224 |
| Phenotype | - | - | - | | |  |  |  | | 2,50.293 | 1.968 | .150 |
| Session×Treatment | 3,92.361 | 1.484 | .224 | | |  |  |  | | 3,121.695 | 1.335 | .266 |
| Session×Phenotype | - | - | - | | |  |  |  | | 6,121.695 | 4.899 | **<.001** |
| Treatment×Phenotype | - | - | - | | |  |  |  | | 2,50.293 | 1.004 | .374 |
| Session×Treatment×Phenotype | - | - | - | | |  |  |  | | 6,121.695 | 0.994 | .433 |
|  | **b. Sessions 5**–**7: Food cup entries during the inter-trial interval** | | | | | | | | | | | |
|  | Treatment | | | |  | | | | Treatment × Phenotype | | | |
|  | DF | *F* | *p* | | |  |  |  | | DF | *F* | *p* |
| Session | 2,97.023 | 15.236 | **<.001** | | |  |  |  | | 2,48.007 | 18.429 | **<.001** |
| Treatment | 1,51.656 | 1.323 | .255 | | |  |  |  | | 1,47.709 | 1.700 | .199 |
| Phenotype | - | - | - | | |  |  |  | | 2,47.719 | 1.153 | .324 |
| Session×Treatment | 2,97.023 | 0.089 | .915 | | |  |  |  | | 2,48.007 | 0.023 | .978 |
| Session×Phenotype | - | - | - | | |  |  |  | | 4,48.007 | 1.765 | .151 |
| Treatment×Phenotype | - | - | - | | |  |  |  | | 2,47.719 | 2.560 | .088 |
| Session×Treatment×Phenotype | - | - | - | | |  |  |  | | 4,48.007 | 3.251 | **.019** |
|  | **c. Sessions 5**–**7: Treatment comparisons within phenotype** | | | | | | | | | | | |
|  | Sign-trackers | | | Intermediate responders | | | | | Goal-trackers | | | |
|  | *p* | | | | | *p* | | | | *p* | | |
| Session 5: VEH vs. CNO | .052 | | | | | .261 | | | | .089 | | |
| Session 6: VEH vs. CNO | .053 | | | | | .971 | | | | .892 | | |
| Session 7: VEH vs. CNO | .061 | | | | | .606 | | | | .703 | | |

***Figure 4-5* Statistical analyses for the effects of treatment and phenotype on food cup entries during the inter-trial interval.**
