# Supplementary material for: Chemogenetic Inhibition of Lateral Hypothalamus Inputs to the Paraventricular Thalamus Reduces Goal-Tracking in a Behaviorally Flexible Subgroup of Male Rats
Source: eNeuro. 2026 Sep 4;13(9):ENEURO.0174-26.2026. doi: 10.1523/ENEURO.0174-26.2026 (PMC13548364; doi:10.1523/ENEURO.0174-26.2026)
Supplement: Supplementary file 3 [file eneuro-13-ENEURO.0174-26.2026-s009.docx]

|  | **a. Session 8: Conditioned Reinforcement** | | | | | | | | | | |
| --- | --- | --- | --- | --- | --- | --- | --- | --- | --- | --- | --- |
|  | Nose Pokes | | |  |  | | |  | Lever Contacts | | |
|  | DF | *F* | *p* |  |  |  |  |  | DF | *F* | *p* |
| Port | 1,52 | 31.889 | **<.001** |  |  |  |  |  | - | - | - |
| Treatment | 1,52 | 0.433 | .514 |  |  |  |  |  | 1,52 | 0.196 | .660 |
| Port×Treatment | 1,52 | 0.053 | .819 |  |  |  |  |  | - | - | - |
|  | **b. Session 8: Conditioned Reinforcement** | | | | | | | | | | |
|  | Nose Pokes | | |  |  | | |  | Lever Contacts | | |
|  | DF | *F* | *p* |  |  |  |  |  | DF | *F* | *p* |
| Port | 1,48 | 44.721 | **<.001** |  |  |  |  |  | - | - | - |
| Treatment | 1,48 | 0.035 | .853 |  |  |  |  |  | 1,48 | 1.282 | .263 |
| Phenotype | 2,48 | 9.221 | **<.001** |  |  |  |  |  | 2,48 | 11.430 | **<.001** |
| Port×Treatment | 1,48 | 0.096 | .758 |  |  |  |  |  | - | - | - |
| Port×Phenotype | 2,48 | 8.537 | **<.001** |  |  |  |  |  | - | - | - |
| Treatment×Phenotype | 2,48 | 0.365 | .696 |  |  |  |  |  | 1,48 | 1.055 | .356 |
| Port×Treatment×Phenotype | 2,48 | 0.623 | .541 |  |  |  |  |  | - | - | - |

***Figure 4-6* Statistical analyses for behavior during the Conditioned Reinforcement Test (CRT).**
