# Supplementary material for: Chemogenetic Inhibition of Lateral Hypothalamus Inputs to the Paraventricular Thalamus Reduces Goal-Tracking in a Behaviorally Flexible Subgroup of Male Rats
Source: eNeuro. 2026 Sep 4;13(9):ENEURO.0174-26.2026. doi: 10.1523/ENEURO.0174-26.2026 (PMC13548364; doi:10.1523/ENEURO.0174-26.2026)
Supplement: Supplementary file 4 [file eneuro-13-ENEURO.0174-26.2026-s010.docx]

|  | Figure 4-7 | | | | | | | | | | | |  |
| --- | --- | --- | --- | --- | --- | --- | --- | --- | --- | --- | --- | --- | --- |
|  | **a. Sessions 1**–**4: Sign-tracking behaviors** | | | | | | | | | | | | |
|  | Lever Contacts | | |  | Probability Lever | | |  | Latency Lever | | | | |
|  | DF | *F* | *p* |  | DF | *F* | *p* |  | DF | *F* | *p* |  |  |
| Session | 3,58 | 10.974 | **<.001** |  | 3,58 | 14.495 | **<.001** |  | 3,87.520 | 12.523 | **<.001** |  |  |
| Treatment | 1,58 | 0.000 | 0.987 |  | 1,58 | 0.078 | 0.781 |  | 1,58.355 | 0.065 | 0.799 |  |  |
| Session×Treatment | 3,58 | 0.316 | 0.814 |  | 3,58 | 0.020 | 0.996 |  | 3,87.520 | 0.170 | 0.916 |  |  |
|  | **b. Sessions 5**–**7: Sign-tracking behaviors** | | | | | | | | | | | | |
|  | Lever Contacts | | |  | Probability Lever | | |  | Latency Lever | | | | |
|  | DF | *F* | *p* |  | DF | *F* | *p* |  | DF | *F* | *p* |  |  |
| Session | 2,72.906 | 0.063 | 0.939 |  | 2, 116 | 0.595 | 0.553 |  | 2, 116 | 0.733 | 0.483 |  |  |
| Treatment | 1,58.163 | 0.035 | 0.851 |  | 1,58 | 0.368 | 0.546 |  | 1,58 | 0.745 | 0.392 |  |  |
| Session×Treatment | 2,72.906 | 0.293 | 0.747 |  | 2, 116 | 0.502 | 0.607 |  | 2, 116 | 0.547 | 0.580 |  |  |
|  | **d. Sessions 1**–**4: Goal-tracking behaviors** | | | | | | | | | | | | |
|  | Food Cup Entries | | |  | Probability Food Cup | | |  | Latency Food Cup | | | | |
|  | DF | *F* | *p* |  | DF | *F* | *p* |  | DF | *F* | *p* |  |  |
| Session | 3,58 | 4.241 | **0.009** |  | 3,80.815 | 5.666 | **0.001** |  | 3,58 | 5.175 | **0.003** |  |  |
| Treatment | 1,58 | 0.005 | 0.943 |  | 1,58.507 | 0.006 | 0.939 |  | 1,58 | 0.015 | 0.904 |  |  |
| Session×Treatment | 3,58 | 0.372 | 0.773 |  | 3,80.815 | 0.204 | 0.893 |  | 3,58 | 0.593 | 0.622 |  |  |
|  | **e. Sessions 5**–**7: Goal-tracking behaviors** | | | | | | | | | | | | |
|  | Food Cup Entries | | |  | Probability Food Cup | | |  | Latency Food Cup | | | | |
|  | DF | *F* | *p* |  | DF | *F* | *p* |  | DF | *F* | *p* |  |  |
| Session | 2,113.482 | 3.402 | **0.037** |  | 2, 76.021 | 0.249 | 0.780 |  | 2,115.032 | 2.262 | 0.109 |  |  |
| Treatment | 1,58.003 | 0.067 | 0.797 |  | 1, 58.036 | 0.018 | 0.893 |  | 1,57.999 | 0.008 | 0.931 |  |  |
| Session×Treatment | 2,113.482 | 1.147 | 0.321 |  | 2, 76.721 | 0.102 | 0.903 |  | 2,115.032 | 0.358 | 0.700 |  |  |

***Figure 4-7.* Statistical analyses for sign- and goal-tracking behaviors in the absence of DREADD.**
