# Supplementary material for: Chemogenetic Inhibition of Lateral Hypothalamus Inputs to the Paraventricular Thalamus Reduces Goal-Tracking in a Behaviorally Flexible Subgroup of Male Rats
Source: eNeuro. 2026 Sep 4;13(9):ENEURO.0174-26.2026. doi: 10.1523/ENEURO.0174-26.2026 (PMC13548364; doi:10.1523/ENEURO.0174-26.2026)
Supplement: Supplementary file 5 [file eneuro-13-ENEURO.0174-26.2026-s011.docx]

|  | Figure 4-8 | | | | | | | | |
| --- | --- | --- | --- | --- | --- | --- | --- | --- | --- |
|  | **a. Sessions 1**–**4: Sign-tracking behaviors** | | | | | | | | |
|  | Lever Contacts | | | Probability Lever | | | Latency Lever | | |
|  | DF | *F* | *p* | DF | F | p | DF | F | p |
| Session | 3,54 | 19.784 | **<.001** | 3,144.296 | 18.010 | **<.001** | 3,95.656 | 23.674 | **<.001** |
| Treatment | 1,54 | 0.019 | 0.890 | 1,55.497 | 0.098 | 0.756 | 1,58.901 | 0.045 | 0.832 |
| Phenotype | 2,54 | 34.867 | **<.001** | 2,55.497 | 62.546 | **<.001** | 2,58.901 | 52.097 | **<.001** |
| Session×Treatment | 3,54 | 1.061 | 0.373 | 3,144.296 | 0.069 | 0.976 | 3,95.656 | 0.208 | 0.891 |
| Session×Phenotype | 6,54 | 16.765 | **<.001** | 6,144.296 | 10.977 | **<.001** | 6,95.656 | 15.878 | **<.001** |
| Treatment×Phenotype | 2,54 | 0.178 | 0.838 | 2,55.497 | 0.368 | 0.694 | 2,58.901 | 0.257 | 0.774 |
| Session×Treatment×Phenotype | 6,54 | 0.625 | 0.710 | 6,144.296 | 0.251 | 0.958 | 6,95.656 | 0.227 | 0.967 |
|  | **b. Sessions 5**–**7: Sign-tracking behaviors** | | | | | | | | |
|  | Lever Contacts | | | Probability Lever | | | Latency Lever | | |
|  | DF | *F* | *p* | DF | *F* | *p* | DF | *F* | *p* |
| Session | 2,108.463 | 0.003 | 0.997 | 2,108 | 0.404 | .669 | 2,108 | 0.713 | .493 |
| Treatment | 1,56.702 | 0.162 | 0.689 | 1,54 | 0.865 | .356 | 1,54 | 1.449 | .234 |
| Phenotype | 2,56.702 | 35.261 | **<.001** | 2,54 | 70.615 | **<.001** | 2,54 | 51.708 | **<.001** |
| Session×Treatment | 2,108.463 | 0.207 | 0.813 | 2,108 | 0.555 | .575 | 2,108 | 0.534 | .588 |
| Session×Phenotype | 4,108.463 | 2.672 | **0.036** | 4,108 | 2.422 | .053 | 4,108 | 2.100 | .086 |
| Treatment×Phenotype | 2,56.702 | 1.076 | 0.348 | 2,54 | 0.349 | .707 | 2,54 | 0.079 | .925 |
| Session×Treatment×Phenotype | 4,108.463 | 0.615 | 0.652 | 4,108 | 0.589 | .671 | 4,108 | 1.599 | .180 |

***Figure 4-8* Statistical analyses for the effects of treatment and phenotype on sign-tracking behaviors in the absence of DREADD.**
