# Supplementary material for: Chemogenetic Inhibition of Lateral Hypothalamus Inputs to the Paraventricular Thalamus Reduces Goal-Tracking in a Behaviorally Flexible Subgroup of Male Rats
Source: eNeuro. 2026 Sep 4;13(9):ENEURO.0174-26.2026. doi: 10.1523/ENEURO.0174-26.2026 (PMC13548364; doi:10.1523/ENEURO.0174-26.2026)
Supplement: Supplementary file 6 [file eneuro-13-ENEURO.0174-26.2026-s012.docx]

|  | Figure 4-9 | | | | | | | | |
| --- | --- | --- | --- | --- | --- | --- | --- | --- | --- |
|  | **a. Sessions 1**–**4: Goal-tracking behaviors** | | | | | | | | |
|  | Food Cup Entries | | | Probability Food Cup | | | Latency Food Cup | | |
|  | DF | *F* | *p* | DF | *F* | *p* | DF | *F* | *p* |
| Session | 3,95.753 | 14.598 | **<.001** | 3,131.599 | 14.093 | **<.001** | 3,107.097 | 14.808 | **<.001** |
| Treatment | 1,57.395 | 0.028 | 0.868 | 1,55.848 | 0.013 | 0.908 | 1,56.370 | 0.000 | 0.990 |
| Phenotype | 2,57.395 | 32.442 | **<.001** | 2,55.848 | 27.536 | **<.001** | 2,56.370 | 25.099 | **<.001** |
| Session×Treatment | 3,95.753 | 0.538 | 0.658 | 3,131.599 | 0.139 | 0.936 | 3,107.097 | 0.594 | 0.620 |
| Session×Phenotype | 6,95.753 | 19.507 | **<.001** | 6,131.599 | 17.863 | **<.001** | 6,107.097 | 18.449 | **<.001** |
| Treatment×Phenotype | 2,57.395 | 0.242 | 0.786 | 2,55.848 | 0.125 | 0.883 | 2,56.370 | 0.049 | 0.952 |
| Session×Treatment×Phenotype | 6,95.753 | 0.880 | 0.513 | 6,131.599 | 0.739 | 0.619 | 6,107.097 | 0.630 | 0.706 |
|  | **b. Sessions 5**–**7: Goal-tracking behaviors** | | | | | | | | |
|  | Food Cup Entries | | | Probability Food Cup | | | Latency Food Cup | | |
|  | DF | *F* | *p* | DF | *F* | *p* | DF | *F* | *p* |
| Session | 2,110.272 | 4.158 | **.018** | 2,71.686 | 0.129 | .879 | 2,104.005 | 2.233 | .112 |
| Treatment | 1,54.017 | 0.148 | .702 | 1,54.109 | 0.294 | .590 | 1,54.001 | 0.146 | .704 |
| Phenotype | 2,54.017 | 46.545 | **<.001** | 2,54.109 | 44.326 | **<.001** | 2,54.001 | 53.707 | **<.001** |
| Session×Treatment | 2,110.272 | 2.122 | .125 | 2,71.686 | 0.131 | .878 | 2,104.005 | 0.492 | .613 |
| Session×Phenotype | 4,110.272 | 1.903 | .115 | 4,71.686 | 0.924 | .455 | 4,104.005 | 0.687 | .602 |
| Treatment×Phenotype | 2,54.017 | 0.238 | .789 | 2,54.109 | 0.493 | .614 | 2,54.001 | 0.159 | .853 |
| Session×Treatment×Phenotype | 4,110.272 | 3.112 | .**018** | 4,71.686 | 1.667 | .167 | 4,104.005 | 1.044 | .388 |
|  | **c. Sessions 5**–**7: Food cup entry effects within phenotype** | | | | | | | | |
|  | Sign-trackers | | | Intermediate responders | | | Goal-trackers | | |
|  | DF | *F* | *p* | DF | *F* | *p* | DF | *F* | *p* |
| Session | 2,38 | 0.452 | .640 | 2,42 | 4.315 | **.020** | 2,28 | 2.243 | .125 |
| Treatment | 1,19 | 0.715 | .408 | 1,21 | 0.022 | .883 | 1,14 | 0.185 | .674 |
| Session×Treatment | 2,38 | 1.643 | .207 | 2,42 | 0.562 | .575 | 2,28 | 2.027 | .151 |

***Figure 4-9* Statistical analyses for the effects of treatment and phenotype on goal-tracking behaviors in the absence of DREADD.**
