# Supplementary material for: Chemogenetic Inhibition of Lateral Hypothalamus Inputs to the Paraventricular Thalamus Reduces Goal-Tracking in a Behaviorally Flexible Subgroup of Male Rats
Source: eNeuro. 2026 Sep 4;13(9):ENEURO.0174-26.2026. doi: 10.1523/ENEURO.0174-26.2026 (PMC13548364; doi:10.1523/ENEURO.0174-26.2026)
Supplement: Supplementary file 7 [file eneuro-13-ENEURO.0174-26.2026-s013.docx]

|  | Figure 4-10 | | | | | | | | | | |
| --- | --- | --- | --- | --- | --- | --- | --- | --- | --- | --- | --- |
|  | **a. Session 8: Conditioned Reinforcement** | | | | | | | | | | |
|  | Nose Pokes | | |  |  | | |  | Lever Contacts | | |
|  | DF | *F* | *p* |  |  |  |  |  | DF | *F* | *p* |
| Port | 1,58 | 22.034 | **<.001** |  |  |  |  |  | - | - | - |
| Treatment | 1,58 | 0.538 | .466 |  |  |  |  |  | 1,58 | 0.832 | .365 |
| Port×Treatment | 1,58 | 0.163 | .687 |  |  |  |  |  | - | - | - |
|  | **b. Session 8: Conditioned Reinforcement** | | | | | | | | | | |
|  | Nose Pokes | | |  |  | | |  | Lever Contacts | | |
|  | DF | *F* | *p* |  |  |  |  |  | DF | *F* | *p* |
| Port | 1,54 | 21.243 | **<.001** |  |  |  |  |  | - | - | - |
| Treatment | 1,54 | 0.990 | .324 |  |  |  |  |  | 1,54 | 0.369 | .546 |
| Phenotype | 2,54 | 3.648 | **.033** |  |  |  |  |  | 2,54 | 10.304 | **<.001** |
| Port×Treatment | 1,54 | 0.095 | .759 |  |  |  |  |  | - | - | - |
| Port×Phenotype | 2,54 | 2.848 | .067 |  |  |  |  |  | - | - | - |
| Treatment×Phenotype | 2,54 | 0.677 | .512 |  |  |  |  |  | 1,54 | 0.062 | .940 |
| Port×Treatment×Phenotype | 2,54 | 0.255 | .776 |  |  |  |  |  | - | - | - |

***Figure 4-10* Statistical analyses for behavior during the Conditioned Reinforcement Test (CRT) in the absence of DREADD.**
